# The residual structure of acid‐denatured β_2_‐microglobulin is relevant to an ordered fibril morphology

**DOI:** 10.1002/pro.4487

**Published:** 2023-01-01

**Authors:** Ryosuke Tomiyama, Masatomo So, Keiichi Yamaguchi, Yohei Miyanoiri, Kazumasa Sakurai

**Affiliations:** ^1^ Graduate School of Biology‐oriented Science and Technology Kindai University Wakayama Japan; ^2^ Institute for Protein Research Osaka University Osaka Japan; ^3^ Astbury Centre for Structural Molecular Biology University of Leeds Leeds UK; ^4^ Global Center for Medical Engineering and Informatics Osaka University Suita Japan; ^5^ High Pressure Protein Research Center, Institute of Advanced Technology Kindai University Wakayama Japan

**Keywords:** amyloid fibril, denatured state, high‐pressure experiment, morphology, NMR, residual structure, β_2_‐microglobulin

## Abstract

β_2_‐Microglobulin (β2m) forms amyloid fibrils *in vitro* under acidic conditions. Under these conditions, the residual structure of acid‐denatured β2m is relevant to seeding and fibril extension processes. Disulfide (SS) bond‐oxidized β2m has been shown to form rigid, ordered fibrils, whereas SS bond‐reduced β2m forms curvy, less‐ordered fibrils. These findings suggest that the presence of an SS bond affects the residual structure of the monomer, which subsequently influences the fibril morphology. To clarify this process, we herein performed NMR experiments. The results obtained revealed that oxidized β2m contained a residual structure throughout the molecule, including the N‐ and C‐termini, whereas the residual structure of the reduced form was localized and other regions had a random coil structure. The range of the residual structure in the oxidized form was wider than that of the fibril core. These results indicate that acid‐denatured β2m has variable conformations. Most conformations in the ensemble cannot participate in fibril formation because their core residues are hidden by residual structures. However, when hydrophobic residues are exposed, polypeptides competently form an ordered fibril. This conformational selection phase may be needed for the ordered assembly of amyloid fibrils.

**Abbreviations and symbols**
[θ]: molar ellipticity
APR: aggregation‐prone region
Aβ: amyloid β
HSQC: heteronuclear single quantum coherence
IDP: intrinsically disordered protein
PC: principal component
PCA: principal component analysis
PRE: paramagnetic relaxation enhancement
*R*_2_
: transverse relaxation rate constant
SS: disulfide
β2m: β_2_‐microglobulin
Δδ: chemical shift perturbation
Δδ_H_
: Δδ values on the ^1^H axis
Δδ_N_
: Δδ values on the ^15^N axis

## 1 INTRODUCTION

Many functional proteins are partially or extensively unfolded and are called intrinsically disordered proteins (IDPs). However, these proteins are not completely unfolded, and they assume a conformational ensemble in which some conformations contain specific structures called residual structures. Ordinary globular proteins may also have residual structures, or a molten globule state, under unfolding conditions, such as in the presence of denaturants or extreme pH.^1^ Since the residual structure is transient, even AI‐driven structural prediction algorithms, which enable highly accurate predictions of native structures of proteins,^2^ cannot precisely predict the properties of residual structures.

Residual structures have been detected in many proteins, including amyloidogenic proteins.^3^, ^4^ Amyloid fibrils are deposited in various organs and tissues throughout the body and cause dysfunctions.^5^ Lysozyme and the variable domain of the IgG light chain have been reported to exhibit residual structures under fibril formation conditions.^6^, ^7^ Furthermore, the polypeptide conformation in prefibrillar oligomeric states is associated with toxicity against cells.^8^ Previous studies demonstrated that some of the oligomeric forms of amyloid β (Aβ) were toxic.^9^, ^10^ These findings indicate that the residual structure is related to the final conformation of proteins, and, thus, further studies on this relationship are needed in order to obtain a more detailed understanding of the pathogenic mechanisms of diseases.

The focus of the present study was β_2_‐microglobulin (β2m). β2m has a typical immunoglobulin fold with a disulfide bond between cysteines at residues 25 and 80. β2m amyloid fibrils are reproducibly formed under acidic pH and a moderate concentration of salt (i.e., 50–100 mM NaCl), which makes it a model system for *in vitro* fibril formation experiments.^11^, ^12^, ^13^, ^14^ Disulfide (SS) bond‐oxidized β2m forms rigid and straight fibrils, whereas SS bond‐reduced β2m forms thin and curvy fibrils.^13^, ^15^ Previous studies reported that acid‐denatured β2m contained a residual structure in which residues assumed interaction sites with the fibril seed in the fibril formation process.^3^, ^4^ Based on these findings, we hypothesized that the presence of an SS bond affects the residual structure of the monomer, which, in turn, influences the fibril morphology.

To elucidate the relationship between the residual structure and fibril morphology, we performed several NMR experiments including a multivariable analysis of pressure‐NMR data, which effectively extracts the residual structure of denatured protein.^16^ The results obtained suggested that, although oxidized and reduced β2m shared a common hydrophobic cluster in 60's residues, the residual structure of the oxidized form was present over a wider range than that of the reduced form. However, not all of the residues involved in the residual structure in the acid‐denatured state constituted the fibril core. The results of paramagnetic relaxation enhancement (PRE) experiments also indicated that the acid‐denatured state assumed a distinct conformation from the final fibril structure. These results suggest that acid‐denatured β2m assumes many conformations. Although hydrophobic residues are covered by the residual structure in the majority of conformations, some have hydrophobic residues that are slightly exposed, which is sufficient to make oriented inter‐chain interactions for the formation of ordered fibrils. This conformational selection phase may be needed for the ordered assembly of amyloid fibrils.

## 2 RESULT

### 2.1 Structural estimation from main‐chain chemical shift data

**FIGURE 1:**
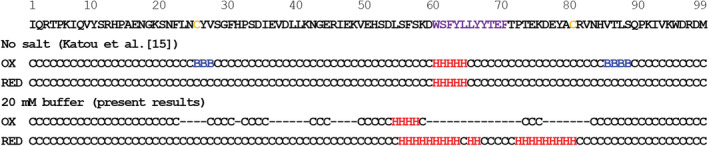
Estimated secondary structures of oxidized and reduced forms from PREDITOR.^17^ Cysteines and 60's hydrophobic residues are colored in yellow and purple, respectively, in the amino acid sequence. H, B, and C in the results indicate the α‐helix, β‐strand, and coil structures, respectively

We initially estimated the secondary structures of acid‐denatured β2m from main‐chain chemical shift data, including ^1^H^α^, ^13^C^α^, ^13^C^β^, and ^13^C′, using PREDITOR.^17^ Standard fibril formation conditions, namely, 100 mM NaCl at pH 2.5, are known to induce the severe signal broadening of β2m, particularly on 60's hydrophobic residues.^18^ Therefore, we performed signal assignment under lower ionic strength conditions (20 mM glycine buffer without salt), and applied the data obtained to estimations (Figure 1 and Table S1). However, significant signal broadening still occurred over the central region for oxidized β2m on 3D NMR spectra. Therefore, we also used the assignment data set of Katou et al.,^15^ in which the solvent condition was only 4 mM HCl and all residues were assigned (Table S2). The measurements of hydrodynamic radius of β2m at pH 2.5 in our previous report^16^ indicated that the oxidized β2m was kept monomer up to 100 mM NaCl and there was no significant protein concentration dependence of the hydrodynamic radius. Thus, the signal broadening is likely attributed to a change in the dynamics of intra‐molecular interactions and not to inter‐molecular interactions.

Figure 1 shows the results of estimations. In the case of the reduced form, α‐helices were detected around the 60's hydrophobic region under no salt conditions, which appeared to be the core of the residual structure. In the presence of 20 mM buffer, the α‐helical region was extended to 55–80, indicating that the residual structure was further formed by the screening or dehydrating effects of the buffer. The salt‐induced enhancement of the secondary structure was also confirmed by CD (Figure S1b): Upon addition of salt, an increase in molar ellipticity ([θ]) at 222 nm and a decrease in [θ] at 205 nm simultaneously observed, consistent with the estimation of the enhancement of the α‐helical content. On the other hand, the oxidized form under no salt conditions showed similar α‐helices around 60's residues. In addition, β‐strands were observed around Cys25 and Cys80, which were likely induced by the SS linkage. In the presence of 20 mM buffer, the signals of many residues showed severe broadening because of the further formation of the secondary structure. However, the β‐sheet estimation around the SS bond disappeared, indicating that the salt‐induced secondary structure was not a simple structural extension, but included a structural rearrangement of the residual structure, which is consistent with our previous findings.^16^ The CD results, an increase in [θ] at 205 nm and no significant change in [θ] at 222 nm upon addition of salt (Figure S1a), again support the picture that there was not a simple enhancement but a conversion of secondary structures. The range of the secondary structure distribution of the oxidized form was wider than that of the reduced form. To further characterize the residual structure of acid‐denatured β2m, we performed pressure unfolding experiments.

### 2.2 Detection of the residual structure as a pressure‐dependent chemical shift perturbation

The structural formation of proteins generally accompanies increases in its molecular volume, causing the destabilization of structured states under high pressure and subsequent pressure unfolding.^19^ The residual structure also unfolds under high pressure, and this unfolding is detected as chemical shift perturbations at the corresponding residues.^16^

**FIGURE 2:**
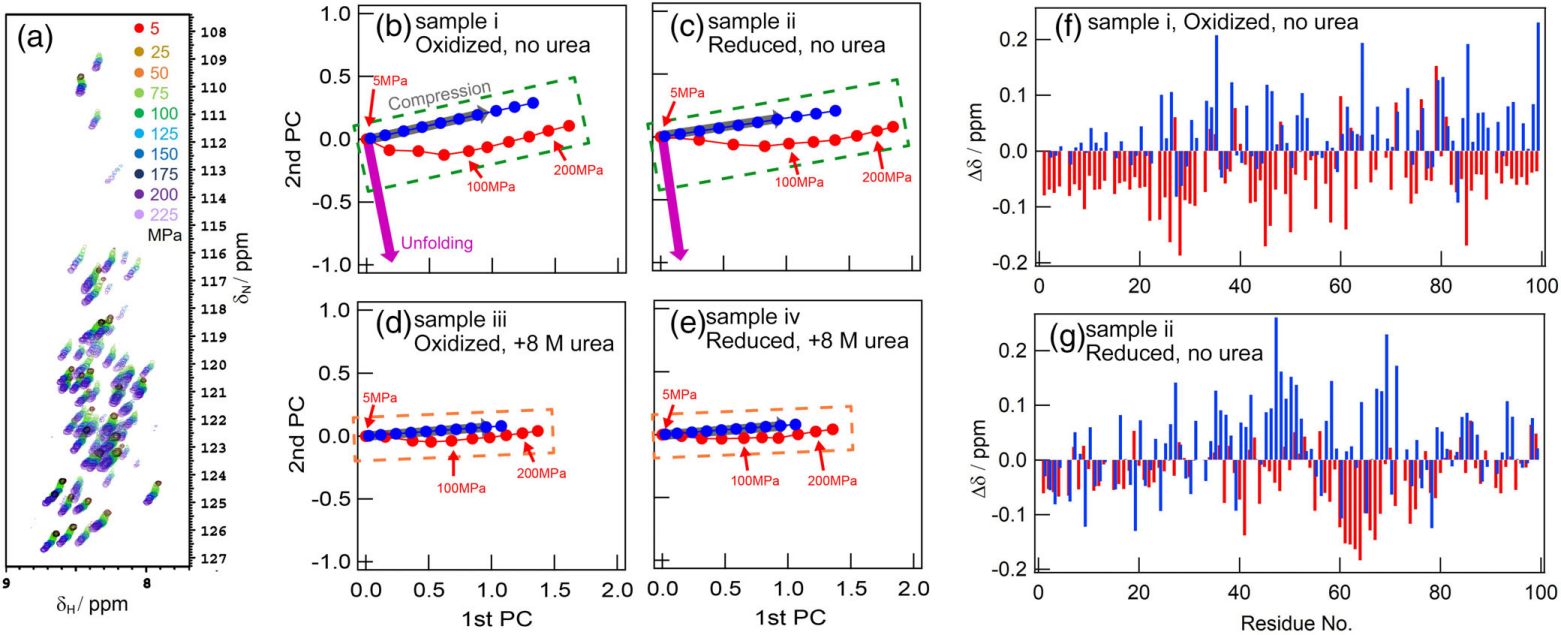
Pressure NMR measurements and PCA results. (a) Superimpositions of ^1^H‐^15^N HSQC spectra at each pressure for oxidized β2m in the absence of urea. The spectral color corresponds to the measurement pressure as indicated. (b‐e) Plot of score values for the first and second principal components. Panels b and c show the results of oxidized and reduced β2m in the absence of urea, respectively. Panels d and e show the results of oxidized and reduced β2m in 8 M urea, respectively. Vertical and horizontal axes represent the score value of the first and second principal components, respectively. The red marker indicates the score value at each pressure point. Blue is a projection of the *b* vector on this plane. Gray and purple arrows indicate the directions corresponding to compression and unfolding, respectively. (f, g) Δδ patterns of the contribution of unfolding. Blue and red lines show chemical shift changes (in ppm) in the ^15^N and ^1^H directions, respectively. The ∆δ values for ^15^N were divided by 5

We monitored the pressure unfolding of oxidized and reduced β2m. As reference data for a completely unfolded state with no residual structure, we also performed the same measurements in the presence of 8 M urea. Figure 2a and Figure S2 show the superposition of heteronuclear single quantum coherence (HSQC) spectra at each pressure. Signal positions changed to lower magnetic fields in the ^1^H and ^15^N axes with increases in pressure. Since proteins “compress” as well as “unfold” in response to pressure, the chemical shift perturbation (Δδ) includes both contributions.^20^ To extract the contribution of unfolding from data, we performed a principal component analysis (PCA) of chemical shift changes.^16^

Table 1 shows the cumulative contributions for the first and second principal component (PC) from the PCA of four samples. Figure S3 shows all of the singular values and cumulative contributions for each sample. The cumulative contribution of the first PC for the sample containing 8 M urea (samples iii and iv) exceeded 90% (Table 1), indicating that their chemical shift data were dependent on only one contribution, namely, compression. On the other hand, the sample without urea (samples i and ii) showed a smaller cumulative contribution ratio for the first PC and a significant increase at the second PC, indicating that their chemical shift data were dependent on two contributions, compression and unfolding.

**TABLE 1:** Cumulative contribution ratio of each sample

Sample	(i) Ox −Urea	(ii) Red −Urea	(iii) Ox +Urea	(iv) Red +Urea
First PC	81.8%	88.4%	91.6%	92.0%
Second PC	88.2%	92.0%	94.6%	94.9%

Figure 2b–e shows plots of score values of the first and second PCs obtained from the PCA (red markers). A projection of the *b* vector is also plotted here (blue markers); the *b* vector is expected to reflect the contribution from the compression of the ideal random coil structure (unfolded structure) based on the *b* value of each amino acid residue reported by Kitahara et al.^20^ The direction along with the projected *b* vector is expected to be the compression direction, and that orthogonal to it is the unfolding direction. Experimental data were interpreted as the linear combination of the compression and unfolding contributions.

The score plots of the sample without urea (samples i and ii, Figure 2b, c) were parallel to the *b* vector at high pressures (~200 MPa), indicating that protein molecules only underwent compression, whereas the plots were curved at low pressures (~100 MPa), suggesting the contributions of both compression and unfolding. The displacement observed along the unfolding direction of the oxidized form (sample i, Figure 2b) was larger than that of the reduced form (sample ii, Figure 2c), indicating a greater contribution from unfolding to the oxidized form.

The score plots of the sample with urea (samples iii and iv, Figure 2d, e) were similar to the *b* vector at all pressures examined. This result indicated that there was only the contribution of compression and no significant contribution of pressure unfolding in the presence of urea, confirming that the polypeptide chain under these conditions contained no residual structure.

Based on the results shown in Figure 2b, c, Δδ values in the unfolding directions for samples i and ii were calculated (Figure 2f, g). The oxidized form showed a negative Δδ_H_ value (Δδ values on the ^1^H axis) throughout the molecule (Figure 2f). Previous studies reported that Δδ_H_ values correlated with the hydrogen bond distance: a positive Δδ_H_ value indicated the formation of an interaction (particularly a hydrogen bond), whereas a negative Δδ_H_ value indicated the breakage of interactions.^21^, ^22^ Therefore, the results obtained indicated the unfolding of the residual structure throughout the molecule upon the application of pressure. The SS bond between the 25th and 80th positions was expected to enhance inter‐residue interactions throughout the molecule even under acid denaturation conditions. Interactions were also detected in the N‐terminal and C‐terminal regions, which is consistent with our previous findings.^16^

In the reduced form, significantly negative Δδ_H_ values were observed around 60's residues, and slightly negative Δδ_H_ values at the residues around 40 (Figure 2g), indicating the local formation of residual structures in these regions. The negative Δδ_H_ values of these residues were larger than those observed for the oxidized form (Figure 2f) because the inherent hydrophobic nature of these residues exclusively contributed to the formation of the local hydrophobic cluster. In addition, a cluster of positive Δδ_N_ values (Δδ values on the ^15^N axis) was observed at residues around 40 (Figure 2g). Since Δδ_N_ values have been reported to correlate with the *ψ* dihedral angle of the preceding residue,^23^, ^24^ this characteristic Δδ_N_ pattern is indicative of cooperative secondary structure formation on these residues. It is noted that the range of the residual structure distribution of the oxidized form was wider than that of the reduced form, which was consistent with secondary structure estimations (Figure 1).

We also calculated the contribution of compression to Δδ values (Figure S4). As expected, all residues showed similar values to *b* values. The Δδ_H_ values obtained were positive under all samples (i–iv), indicating the shrinkage of the entire molecule because of compression. Δδ_H_ and Δδ_N_ in the absence of urea (Figure S4a, b) were similar to those in the presence of urea (Figure S4c, d), which may have been because the main conformation for all samples was the unfolded state and the conformation harboring a residual structure was only slightly populated.

### 2.3 Transverse relaxation rate constant measurements

**FIGURE 3:**
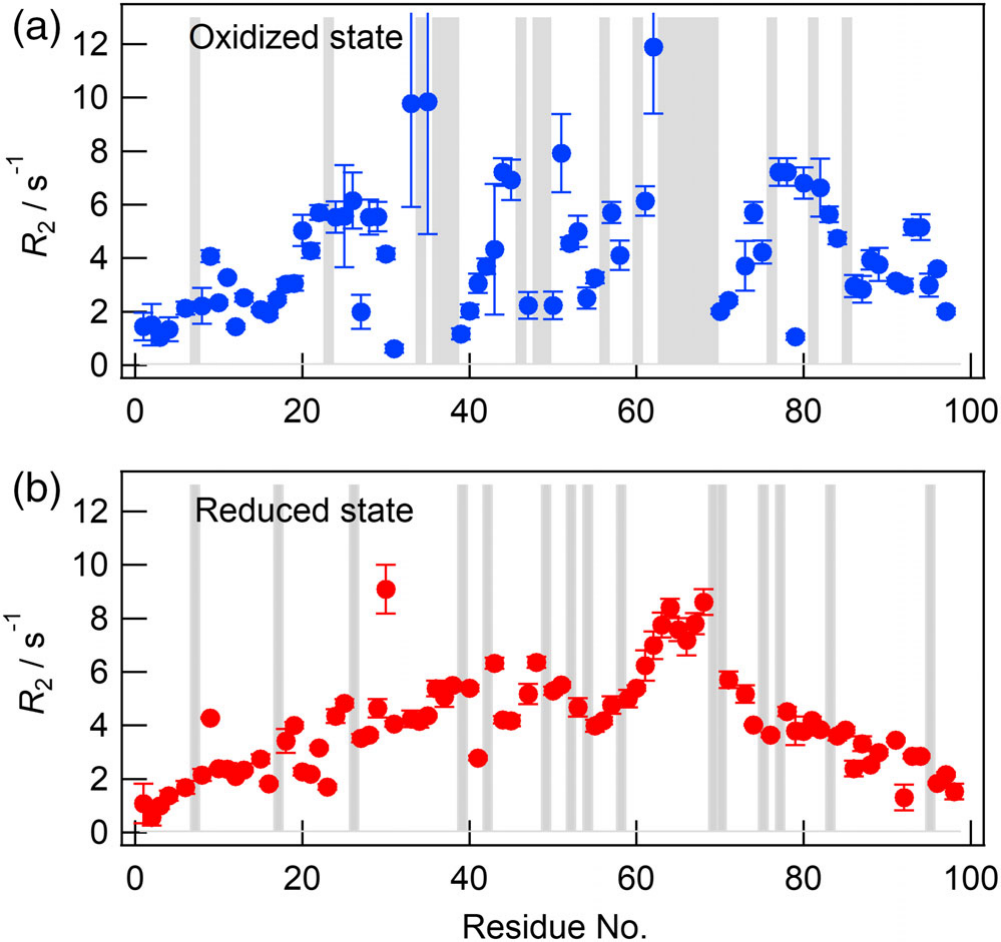
Transverse relaxation rate constant (*R*_2_) of each residue. The results for the *R*_2_ value of each residue of the oxidized (a) and reduced (b) forms are shown. Areas filled in gray indicate residues for which a signal was not identified

To obtain insights into flexibility, the transverse relaxation rate constant (*R*_2_) of each residue was measured (Figure 3). In the oxidized form, residues 20–80 had higher *R*_2_ values than the ends (Figure 3a). No signal was observed at residues 32–39 or 60–70, which may have been due to the markedly higher relaxation rate.^3^ On the other hand, in the reduced form, signals were basically observed from all residues. Although *R*_2_ of residues 60–70 was relatively high, the pattern was generally convex and smooth (Figure 3b).

Regarding oxidized forms, the *R*_2_ values at the N‐ and C‐terminal residues were lower than at the central residues, which is in contrast to the results of PCA. This discrepancy may be attributed to differences in measurement sensitivity depending on the timescale of residual interactions: the formation of residual structures at the N‐ and C‐terminal regions appeared to occur on a fast (ns order) timescale.

In comparison with the reduced form, the oxidized form has decreased mobility around Cys25 and Cys80 due to the residual structure induced by the SS bond. The *R*_2_ values of residues 39–43 of the oxidized form were less than those of the reduced form, indicating that these residues were free from residual interactions. Since the SS bond may restrict mobility over the molecule, these lower *R*_2_ values are counter‐intuitive. The SS linkage appeared to release the restriction of mobility of these residues.

### 2.4 Paramagnetic relaxation enhancement

**FIGURE 4:**
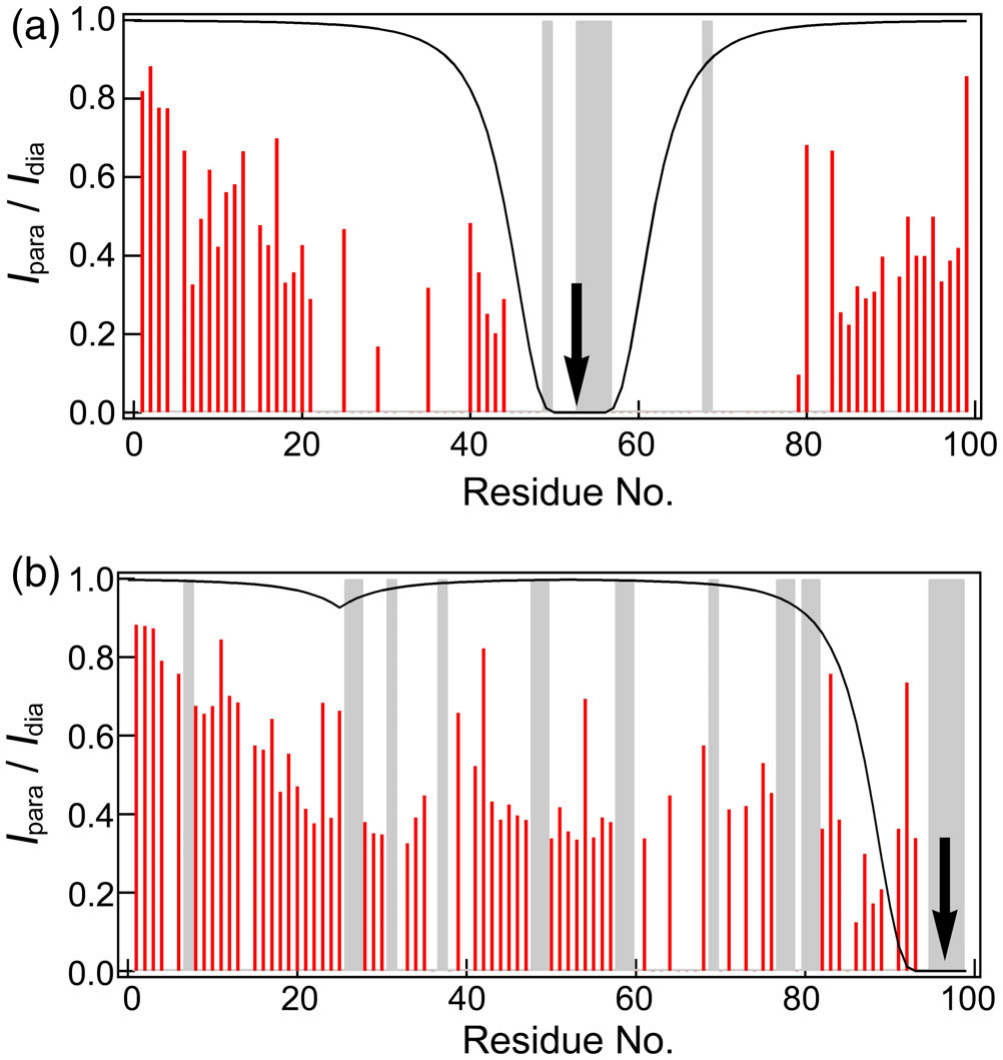
Results of paramagnetic relaxation enhancement (PRE) experiments of 53‐labeled (a) and 96‐labeled (b) β2m in the oxidized form. The red line represents the relative signal intensities of the spin‐labeled sample with respect to those of the diamagnetic reference sample. The black line shows the predicted value assuming a random coil structure. Arrows indicate the positions of the spin labels. Areas filled in gray indicate residues for which a signal was not identified even in the diamagnetic sample

PRE experiments were performed to obtain information on inter‐residue distances (Figure 4). We attached spin labels at the 53rd or 96th positions of oxidized β2m. In both samples, many residues showed lower intensities than those predicted for random coils. In 53‐labeled β2m, the N‐ and C‐terminal regions also showed lower values than predicted. These results indicated that these regions were close to the central part of the sequence, which was consistent with the results of pressure NMR (Figure 2f). Furthermore, the signal intensity significantly decreased around Cys25 and Cys80. The signal intensities of residues 40–45 remained, whereas those of residues adjacent to this region almost disappeared (Figure 4a). These results suggested that the region around 40 and 53 had fewer residual interactions between them, which was consistent with *R*_2_ results. In 96‐labeled β2m, signal intensity decreased by 50% on residues ranging from the 20th position to the C terminus (Figure 4b).

We also spin‐labeled reduced β2m (Figure S5). However, since spin reagents were reacted after the reduction of the SS bond, the inherent cysteine residues (Cys25 and Cys80) were also modified, generating triple‐label species. Although these species showed larger decreases in intensity than their oxidized counterparts, quantitative interpretations were challenging and, thus, we did not discuss these data.

## 3 DISCUSSION

### 3.1 Features of the residual structure of acid‐denatured β2m

**FIGURE 5:**
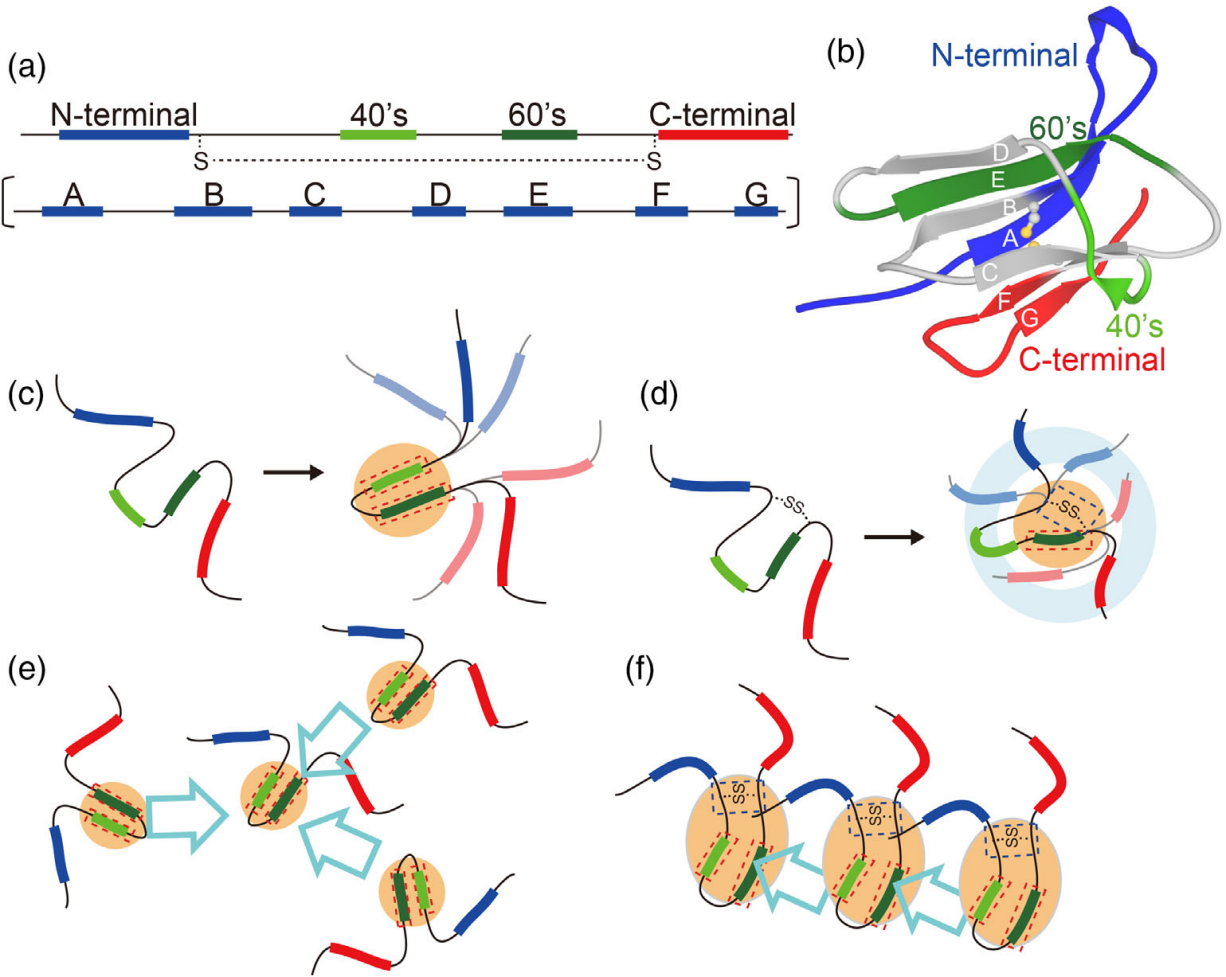
Proposed mechanism for the formation of (less‐)ordered fibrils of β2m. (a) Schematic representations of structural elements responsible for fibril formation. (b) The positions or the N‐terminal region (blue), 40's residues (light green), 60's residues (green), and C‐terminal region (red) indicated on the native structure of β2m. (c, d) Proposed models for the residual structures of monomers for the reduced form (c) and oxidized form (d). Orange areas indicate hydrophobic cluster regions. Light blue areas and orange areas indicate the region at which residual structures formed. (e, f) Illustrations of the orientations of the inter‐monomer associations for the reduced form (e) and oxidized form (f)

The structural characterization of β2m revealed that 60's residues (Figure 5a,b, green region) were relevant to the formation of the residual structure in both the oxidized and reduced forms, whereas other regions, that is, the N‐ and C‐terminal regions (Figure 5a,b, blue and red regions, respectively) and 40's residues (Figure 5a,b, light green region) showed distinct behaviors between them. These differences likely contributed to the difference in the morphology, or macroscopic appearances, of the fibrils between the oxidized and reduced forms.

β2m forms amyloid fibrils under acidic pH in the presence of a moderate concentration of salt, that is, 50–100 mM NaCl. Under these conditions, the 60's residues of reduced β2m were suggested to form a local α‐helix and attached to 40's residues through hydrophobic interactions, which made a residual structure (Figure 5c, orange). In the case of the oxidized form, 60's residues were also part of the residual structure and the SS bond directly induced β‐sheet formation near Cys25 or Cys80. Furthermore, this SS bond restricted the mobility of the polypeptide chain, which caused residual interactions over the entire molecule with a certain rearrangement (Figure 5d, orange). However, 40's residues assumed a more random‐coil‐like conformation in the oxidized form (Figure 5d, light green strand).

An interesting behavior observed in the present study was the α‐β transition; it was originally reported in the non‐native α‐helical conformation in the refolding intermediate of the β‐rich protein.^25^ The formation of α‐helical structures in prefibrillar states was also reported for α‐synuclein^26^ and Aβ.^27^ This phenomenon is observed when a conversion from a short‐range to long‐range hydrophobic interaction is relevant,^28^ with the former and latter contributing to α‐helical and β‐sheet structures, respectively.

The oxidized and reduced forms both showed the expansion of the range of secondary structure regions following an increase to a moderate ionic strength. The screening of electrostatic repulsion or dehydration by salt strengthened hydrophobic interactions, leading to conformations with more secondary structures. This effect was stronger for the oxidized form than for the reduced form due to the restriction by the SS bond. The NMR signals of some residues were unassigned because of severe broadening in 3D NMR spectra, which was caused by a greater conformational change occurring in a μs‐ms timescale. However, the residual structure of oxidized β2m under the moderate salt concentration was not formed through a simple increase in the secondary structure region, but by an alteration in the conformation from the low salt conformation.

It is known that *cis*/*trans* conformational changes at Pro32 is relevant to amyloid fibril formation in the physiological conditions.^29^, ^30^, ^31^, ^32^ On the other hand, all prolines in denatured β2m are supposed to prefer a *trans* conformation.^30^, ^31^, ^32^ Since two observable prolines, Pro32 and Pro72, in the cryo‐EM structure of amyloid fibrils formed from acid‐denatured β2m also assume *trans* conformations,^12^ it is suggested that oxidized β2m undergoes conformational changes into fibril structure with the prolines kept *trans* conformations. According to literatures, the ^13^C^β^ chemical shift tends to show downfiled shifts upon a *trans* to *cis* conformational change.^33^, ^34^ However, the present assignment results (Table S1) showed no significant changes in the proline ^13^C^β^ chemical shifts between oxidized and reduced forms, indicating that proline conformations seem to be irrelevant to the monomeric conformations in the acid‐denaturation conditions.

### 3.2 Differences between the residual structure of the monomer and the final fibril structure in the oxidized form

It is interesting that the final fibril structure slightly differed from the residual structure of the monomer in the oxidized form. The low *R*_2_ values (Figure 3) and high relative intensities in PRE measurements (Figure 4) around 40 indicated that these residues assumed a random coil behavior. However, the recently reported cryo‐EM‐based structure indicated that these residues were involved in the core structure and never assumed a random coil conformation.^12^ Therefore, during the nucleation step, conformational alterations occurred at least in these regions. In the monomeric form, many conformations were sampled and some were selected to form the final fibril structure. The light chain fragment of antibody was previously reported to undergo gradual conformational changes in the prefibrillar phase.^35^

### 3.3 Conditions for ordered fibril formation

We herein discuss how the residual structure affects the morphology of fibrils. When association orientations between monomers are fixed, ordered fibrils are likely to be formed. In the reduced form, 40's and 60's residues, the key regions for monomer‐monomer associations, had a local residual structure. The other regions assumed a random coil structure, which had negligible effects on this association (Figure 5c). Under these conditions, various association orientations may occur, resulting in the formation of less‐ordered fibrils (Figure 5e). On the other hand, oxidized β2m formed a residual structure not only in 60's hydrophobic regions but also on the entire molecule. Residual structures at the residues not involved in the hydrophobic regions were assumed to cover association spots (Figure 5d, light blue), inhibiting the inter‐monomer association through hydrophobic residues. However, the polypeptide chain sampled various conformations, some of which had hydrophobic residues that were slightly accessible to the solvent. The exposure of a small part of hydrophobic residues enabled associations in a specific orientation between monomers. The residual β‐structures around the Cys25 and Cys80 (Figure 1) also contribute to the association orientation. Although the β‐structures were estimated to disappear in the presence of buffer, these regions will assume β‐structure upon inter‐molecular association due to an inherent preference, as observed in the cryo‐EM structure.^12^ These events simultaneously occur upon inter‐monomer association, leading to aligned and rigid fibrils (Figure 5f).

These pictures clearly explain the existence of the lag phase in the nucleation step. Reduced β2m formed less‐ordered fibrils without a clear lag phase,^36^ that is, there was no clear nucleation phase. On the other hand, the spontaneous fibril formation of the oxidized form had a typical lag phase.^18^ Based on the discussion above, the lag phase is a sampling period for conformations with exposed hydrophobic core regions and those that enable monomer‐monomer associations with a specific orientation. Two or more of these species should simultaneously emerge and encounter each other. Furthermore, such an associate should assume an appropriate conformation for a template, which takes time.

One possibility is that the initial association occurred at the residues around 40 because these residues assumed random conformations and were expected to be exposed to the solvent compared to other regions (Figure 5d, light green strand). This result is consistent with previous findings by Muta et al.^18^ showing that the K3 peptide, which shared residues around 40, weakly and less specifically interacted with full‐length β2m in the prefibrillar phase.

### 3.4 Effects of disulfide bonds on polymorphisms

Having the main chain structure of protein has been recognized to be the dominant factor to assume amyloid fibril structures.^36^ However, the recently reported atomistic structures of amyloid fibrils revealed by cryo‐EM and solid‐state NMR indicated that side‐chain packing is also important,^37^ like the native states of proteins.^38^ Multiple kernel structures of amyloid fibrils have been reported for some IDPs, such as α‐synuclein^39^, ^40^, ^41^, ^42^, ^43^ and Aβ.^44^, ^45^, ^46^, ^47^, ^48^ The immunoglobulin light chain, a globular protein, has also been reported to form several fibril structures.^49^, ^50^, ^51^, ^52^ In these proteins and peptides, the existence of many aggregation‐prone regions (APRs) was estimated from their sequences and they were widespread over the sequence (Figure S6b‐f). These results indicate that multiple combinations of intramolecular interactions between these regions generate the observed multiplicity in the kernel structure. On the other hand, β2m, which is also an immunoglobulin, only had two localized APRs around the 20 and 60 positions (Figure S6a). In addition, its polypeptide conformation was restricted by the SS bond. With these properties, when oxidized β2m forms an ordered fibril, only a specific kernel structure was favored, as evidenced by the limited number of studies on the atomistic structure of β2m to date.^12^ On the other hand, the less‐ordered fibril morphology for reduced β2m revealed by AFM images indicate that the fibrils lack a defined kernel structure.^13^, ^15^ This is attributed to the increased flexibility of the peptide chain, in which the specific stacking of hydrophobic core regions rarely occurs.

In the folding process, particularly from the molten globule state to the final native structure, correct associations between the local folding elements are essential. SS bonds contribute to these associations. Even in amyloid fibril formation, SS bonds play a similar role in restricting specific associations between APRs, which leads to an ordered fibril structure. However, the SS bond also contributes another property to the polypeptide; the SS bond linkage gathers amyloid‐irrelevant regions (N‐ and C‐terminal regions in this case), which hinder intermolecular hydrophobic interactions. This effect causes the general lag phase behavior in which aggregation‐competent structures and orientations are examined. Both of these properties of the SS bond contribute to the ordered fibril morphology.

### 3.5 Concluding remarks

In the present study, we attempted to characterize the residual structure of acid‐denatured β2m by various NMR measurements and data analyses of chemical shift data. The measurements under these conditions focused on the features of residual structure relevant to the inter‐molecular association in the initial stage of the fibril formation. The results obtained revealed that oxidized and reduced β2m assumed distinct residual structures; in oxidized β2m, the N‐ and C‐terminal regions, which are involved in the residual structures but not in the hydrophobic regions, covered the association spots. A small proportion of species with a limited area of exposure in hydrophobic regions associated with each other in a specific orientation, leading to the occurrence of the lag phase and the subsequent formation of ordered fibrils. On the other hand, since the residual structure of the reduced form was localized, there were fewer restrictions on the intermolecular orientation, resulting in the formation of less‐ordered fibrils without a lag phase.

The present results are important for obtaining a more detailed understanding of the factors influencing fibril morphology. Furthermore, the analytical methods performed herein are effective for other systems in which the residual structure is relevant to molecular assembly, such as liquid–liquid phase separation phenomena.

## 4 MATERIALS AND METHODS

### 4.1 Expression, refolding, and purification of recombinant β2m in the oxidized form

β2m was expressed by the *Escherichia coli* BL21 strain with a pCold IV plasmid harboring the β2m gene. The transformant was cultured in M9 synthetic medium with vigorous shaking at 37°C until OD600 = 0.5. IPTG was added and the culture was continued at 16°C for 24 hr to induce expression. Bacteria were harvested by centrifugation. Inclusion bodies containing expressed β2m were purified using BugBuster (Merck). The inclusion bodies obtained were dissolved in 8 M urea solution containing 20 mM Tris–HCl (pH 8.0). The protein solution was stirred at 4°C for 3 days for a complete formation of the intramolecular SS bond by air oxidization, and then dialyzed against 20 mM Tris–HCl (pH 7.2) to remove urea. The solution obtained was applied to a DEAE FF column (GE Healthcare). The adsorption buffer was 20 mM Tris–HCl (pH 7.5), and elution was performed with a NaCl concentration gradient from 0 to 500 mM. The eluted fraction was dialyzed against ion‐exchanged water and lyophilized.

### 4.2 Preparation of reduced β2m

Lyophilized β2m was dissolved in 20 mM Tris–HCl (pH 8.0), 8 M urea, and 10 mM dithiothreitol at room temperature for 30 min. The buffer was replaced with 2.8 mM HCl on a PD‐10 column (Cytiva) and the protein solution was lyophilized. We confirmed by AFM that reduced β2m prepared in this way showed thin and curvy fibrils as expected (Figure S7a). It is a clear contrast to that oxidized β2m showed rigid and straight fibril (Figure S7b).

### 4.3 Chemical shift assignments and secondary structure estimations

NMR measurements were performed using a Bruker Avance III 600 MHz NMR apparatus equipped with a Z‐axis gradient and triple resonance TXI probe (Bruker). HNCACB, CACBCONH, HNCO, HNCACO, and TOCSY‐HSQC spectra were measured for chemical shift assignments under our experimental condition of 20 mM Gly (pH 2.5) at 25°C (Table S1). Protein concentrations were 6.1 mg/mL for oxidized β2m and 9.9 mg/ml for reduced β2m.

Secondary structures were estimated from the chemical shift data, including ^1^H^α^, ^13^C^α^,^13^C^β^, and ^13^C′, of oxidized and reduced β2m using PREDITOR.^17^ We used our chemical shift data and those reported by Katou et al.^15^ as input data (Table S2). It is noted that the chemical shift data obtained above was only used for the secondary structure estimations.

### 4.4 Pressure NMR measurements

Sample solutions for high‐pressure NMR measurements were 4.0 mg/mL of oxidized or reduced β2m, 20 mM Gly (pH 2.5), 0.04 mM DSS, and 10% D_2_O in the presence or absence of 8 M urea. The sample solution was placed in a homemade ceramic cell connected online to an external pump.^53^ ^1^H‐^15^N HSQC measurements were performed at 5, 25, 50, 75, 100, 125, 150, 175, 200, and 225 MPa, with 5 MPa being set as the ambient pressure to suppress bubble formation. NMR data were processed using nmrPipe^54^ and analyzed using SPARKY (Goddard and Kneller, SPARKY 3, University of California, San Francisco). Some signals for oxidized β2m were observable on ^1^H‐^15^N HSQC spectra but were unassigned by the sequential assignment performed above. Therefore, the ^1^H^N^ and ^15^N^H^ chemical shifts of unassigned residues were estimated based on similarities to those obtained under no salt conditions (Table S1).

Using chemical shift data, a matrix **X** was created in which each pressure point was in the column and each amino acid residue was in the row. The matrix size was 148 [74 (number of traceable residues) × 2 (δ_H_ and δ_N_)] × 10 (different pressure points). Singular value decomposition on **X** was performed to obtain singular values for each PC. Details of the analysis are described in our previous study.^56^

### 4.5 Transverse relaxation measurement

Measurements of the *R*_2_ values of ^15^N nuclei in backbone amides were conducted using the pulse sequence described by Farrow et al.^57^ Samples were 0.8 mg/ml oxidized or reduced β2m, 20 mM Gly (pH 2.5), and 10% D_2_O. The experiment included a series of 10 experiments with transverse decay times ranging between 17.6 and 176 ms. Data were processed and analyzed for the *R*_2_ values of each residue using nmrPipe and SPARKY.

### 4.6 PRE measurements

A total of 2 mg/mL D53C or D96C β2m containing 20 mM Tris–HCl (pH 8.0) and 1.5 mM Tris(2‐carboxyethyl)phosphine was incubated for 1 hr. 4‐Maleimido‐TEMPO was then added and incubated for 4 hr. The solution was divided into two. In diamagnetic reference samples, we added ascorbic acid (final concentration of 10 mM) to one of the solutions and incubated it for 1 hr. Unreacted reagents were removed with a PD‐10 column by replacing the buffer with 20 mM Gly. Protein samples were concentrated to 1.2 mg/ml by ultrafiltration.

After measurements of HSQC spectra, the signal intensity of paramagnetic‐labeled samples relative to the signal strength of the diamagnetic reference, *I*_para_/*I*_dia_, was calculated. *I*_para_/*I*_dia_ values for an ideal random coil were also calculated using Equation (1).

(1) $$\frac{I_{\text{para}}}{I_{\mathrm{dia}}}=\frac{R_{2,\mathrm{dia}}\exp\left(-R_{2P}t\right)}{R_{2,\mathrm{dia}}+R_{2P}}$$

The derivation of Equation (1) and the following calculation for the predicted *I*_para_/*I*_dia_ ratio are described in Supplemental Information.

## AUTHOR CONTRIBUTIONS

**Ryosuke Tomiyama:** Formal analysis (equal); investigation (equal); methodology (equal); writing – original draft (equal). **Masatomo So:** Conceptualization (equal); resources (equal); writing – review and editing (equal). **Keiichi Yamaguchi:** Methodology (equal); writing – review and editing (equal). **Yohei Miyanoiri:** Methodology (equal); writing – review and editing (equal). **Kazumasa Sakurai:** Conceptualization (lead); data curation (equal); funding acquisition (lead); investigation (equal); supervision (lead); writing – review and editing (lead).

## Supporting information


**Figure S1.** CD spectral changes in acid‐denatured β2m at various ionic strengths.
**Figure S2.** Results of pressure NMR measurements.
**Figure S3.** The contribution ratio and cumulative contribution ratio of each principal component for respective samples.
**Figure S4.** Δδ patterns of the contribution of compression obtained from PCA.
**Figure S5.** Results of paramagnetic relaxation enhancement experiments.
**Figure S6.** Predictions of aggregation‐prone regions.
**Figure S7.** AFM images of amyloid fibrils of reduced and oxidized β2m.
**Supplementary Method.** Deviation of *I*_para_/*I*_dia_ values for random coils.
**Table S1.** Polypeptide backbone ^1^H, ^13^C, and ^15^ N chemical shifts for β2m in the acid‐denatured state at 20 mM Gly‐HCl (pH 2.5) and 25°C.
**Table S2.** Polypeptide backbone ^1^H, ^13^C, and ^15^ N chemical shifts for β_2_‐microglobulin in the acid‐denatured state at 4 mM HCl (pH 2.5) and 37°C reported by Katou et al.

## Data Availability

Data available on request from the authors.
