# Supplementary material for: The residual structure of acid‐denatured β2‐microglobulin is relevant to an ordered fibril morphology
Source: Protein Sci. 2023 Jan 1;32(1):e4487. doi: 10.1002/pro.4487 (PMC9793977; doi:10.1002/pro.4487)
Supplement: Supplementary file 1 — Figure S1. CD spectral changes in acid‐denatured β2m at various ionic strengths. Figure S2. Results of pressure NMR measurements. Figure S3. The contribution ratio and cumulative contribution ratio of each principal component for respective samples. Figure S4. Δδ patterns of the contribution of compression obtained from PCA. Figure S5. Results of paramagnetic relaxation enhancement experiments. Figure S6. Predictions of aggregation‐prone regions. Figure S7. AFM images of amyloid fibrils of reduced and oxidized β2m. Supplementary Method. Deviation of I para/I dia values for random coils. Table S1. Polypeptide backbone 1H, 13C, and 15 N chemical shifts for β2m in the acid‐denatured state at 20 mM Gly‐HCl (pH 2.5) and 25°C. Table S2. Polypeptide backbone 1H, 13C, and 15 N chemical shifts for β2‐microglobulin in the acid‐denatured state at 4 mM HCl (pH 2.5) and 37°C reported by Katou et al. [file PRO-32-e4487-s001.pdf]

## Supplementary Materials for

# The residual structure of acid-denatured $\beta_2$-microglobulin is relevant to an ordered fibril morphology

*Ryosuke Tomiyama[1], Masatomo So[2,3], Keiichi Yamaguchi[4], Yohei Miyanoiri[2], and Kazumasa Sakurai[1,5,*]*

[1]Graduate School of Biology-oriented Science and Technology, Kindai University, 930 Nishimitani, Kinokawa, Wakayama 649-6493, Japan, [2]Institute for Protein Research, Osaka University, 3-2 Yamadaoka, Suita, Osaka 565-0871, Japan, [3]Astbury Centre for Structural Molecular Biology, University of Leeds, Leeds, LS2 9JT, UK, [4]Global Center for Medical Engineering and Informatics, Osaka University, 2-1 Yamadaoka, Suita 565-0871, Japan, [5]High Pressure Protein Research Center, Institute of Advanced Technology, Kindai University, 930 Nishimitani, Kinokawa, Wakayama 649-6493, Japan.

*Corresponding author: Kazumasa Sakurai, E-mail: sakurai@waka.kindai.ac.jp; phone: +81-736-77-0345 (ex. 5004)

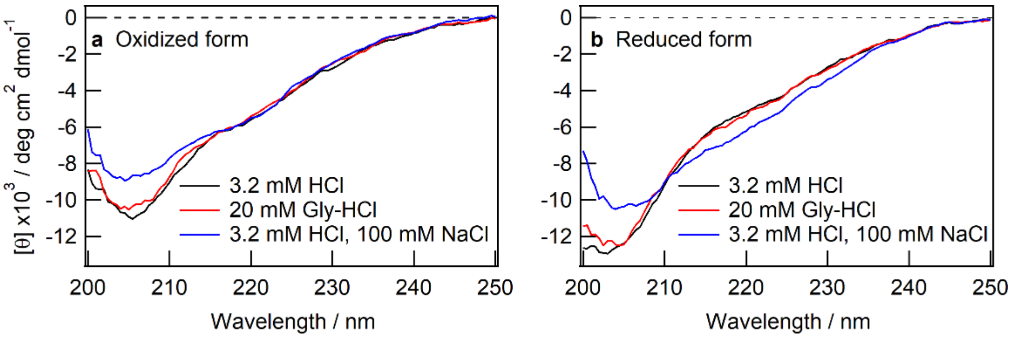


**Figure S1.** CD spectral changes in acid-denatured β2m at various ionic strengths.

CD spectra obtained under 3.2 mM HCl (black), 20 mM Gly (red), and 3.2 mM HCl and 100 mM NaCl (blue) for (a) oxidized β2m and (b) reduced β2m are shown.

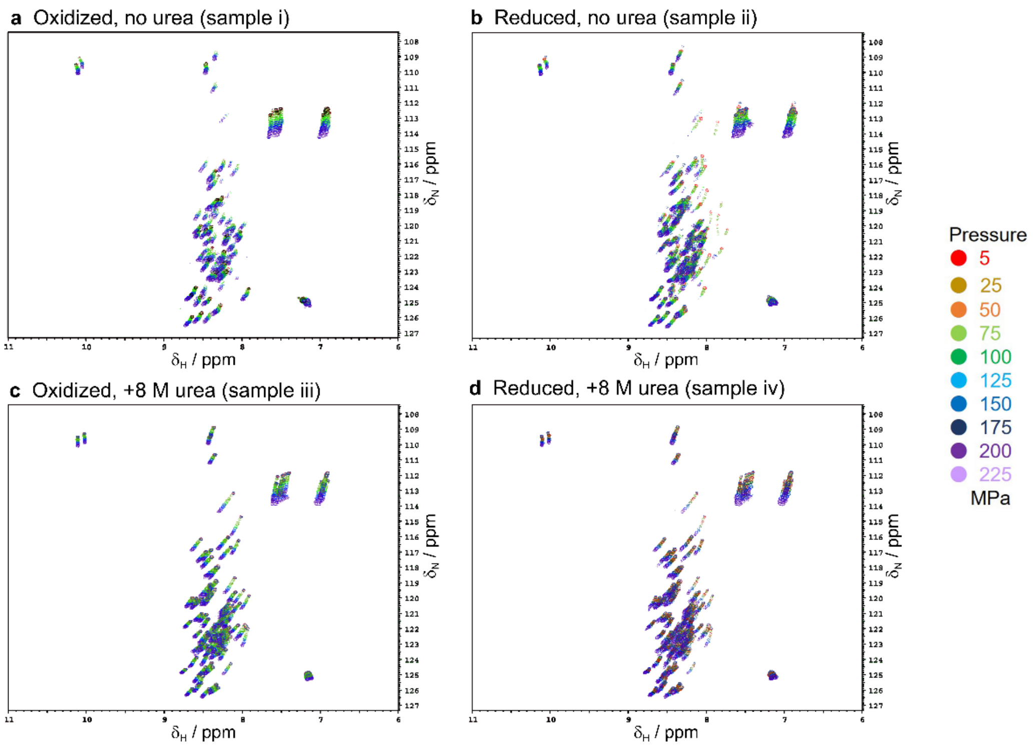


**Figure S2**. Results of pressure NMR measurements.

Superimpositions of $^{1}H$-$^{15}N$ HSQC spectra at each pressure point for (a) oxidized β2m in the absence of urea, (b) reduced β2m in the absence of urea, (c) oxidized β2m under 8 M urea, and (d) reduced β2m under 8 M urea are shown. The spectral color corresponds to the measurement pressure as indicated on the right side of the figure.

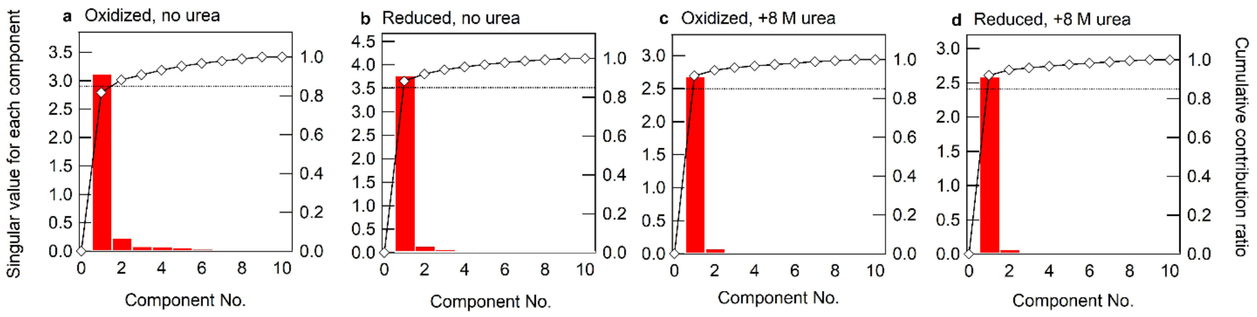


**Figure S3.** The contribution ratio (diamond) and cumulative contribution ratio (red bars) of each principal component for respective samples.

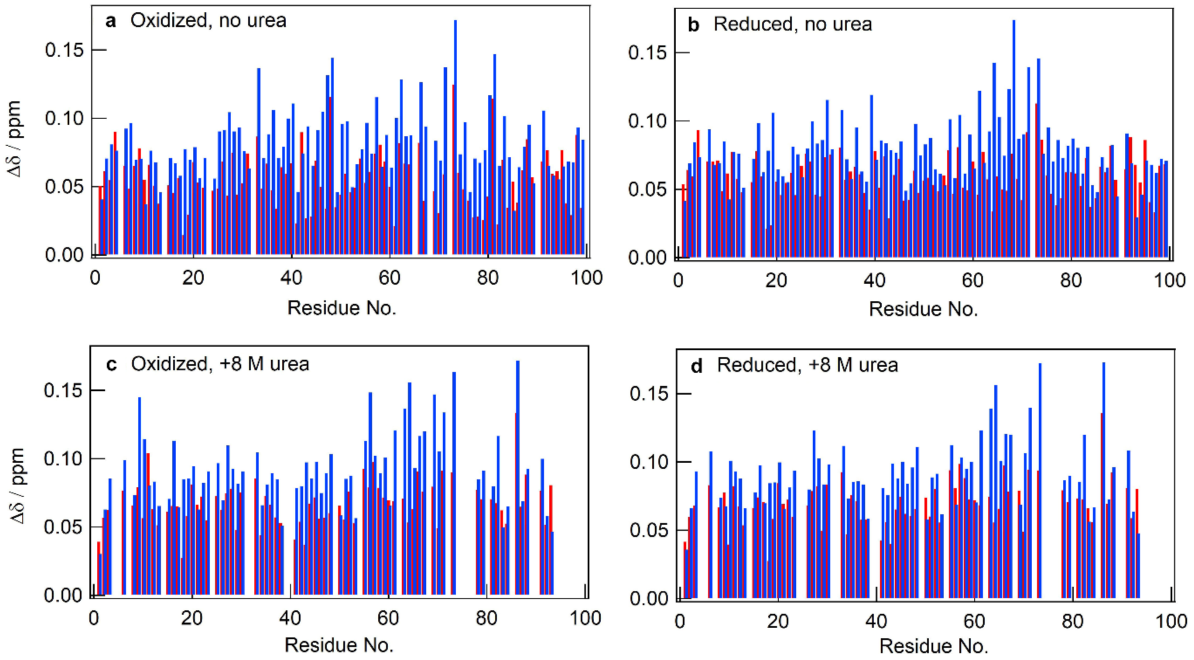


**Figure S4.** Δδ patterns of the contribution of compression obtained from PCA.

The results of (a) oxidized β2m in the absence of urea, (b) reduced β2m in the absence of urea, (c) oxidized β2m under 8 M urea, and (d) reduced β2m under 8 M urea, are shown. Blue and red bars represent Δδ of nitrogen ($\Delta\delta_N$) and hydrogen ($\Delta\delta_H$), respectively. Δδ of nitrogen is divided by 5.

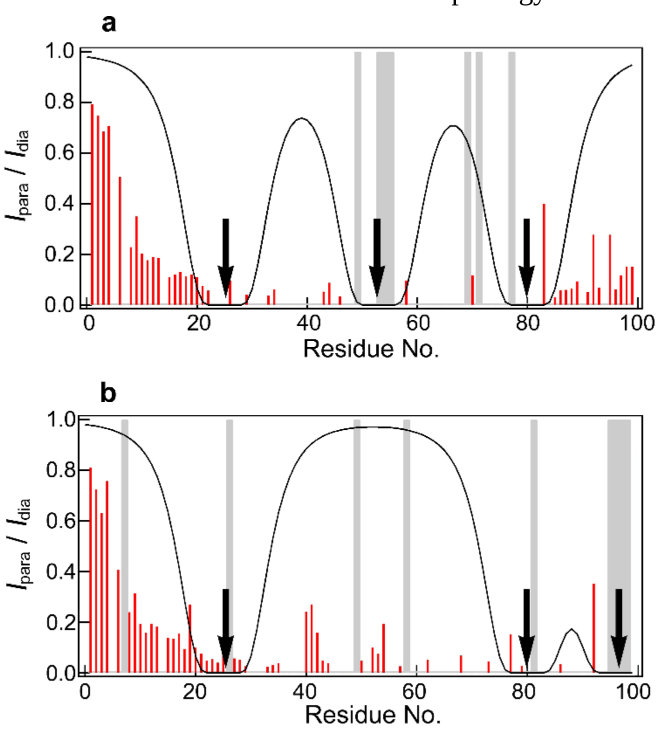


**Figure S5.** Results of paramagnetic relaxation enhancement (PRE) experiments on 53-labeled (a) and 96-labeled (b) β2m in the reduced form.

The red line represents the relative signal intensities of the spin-labeled sample with respect to those of the diamagnetic reference sample. The black line shows the predicted value assuming a random coil structure. The arrows indicate the positions of the spin labels.

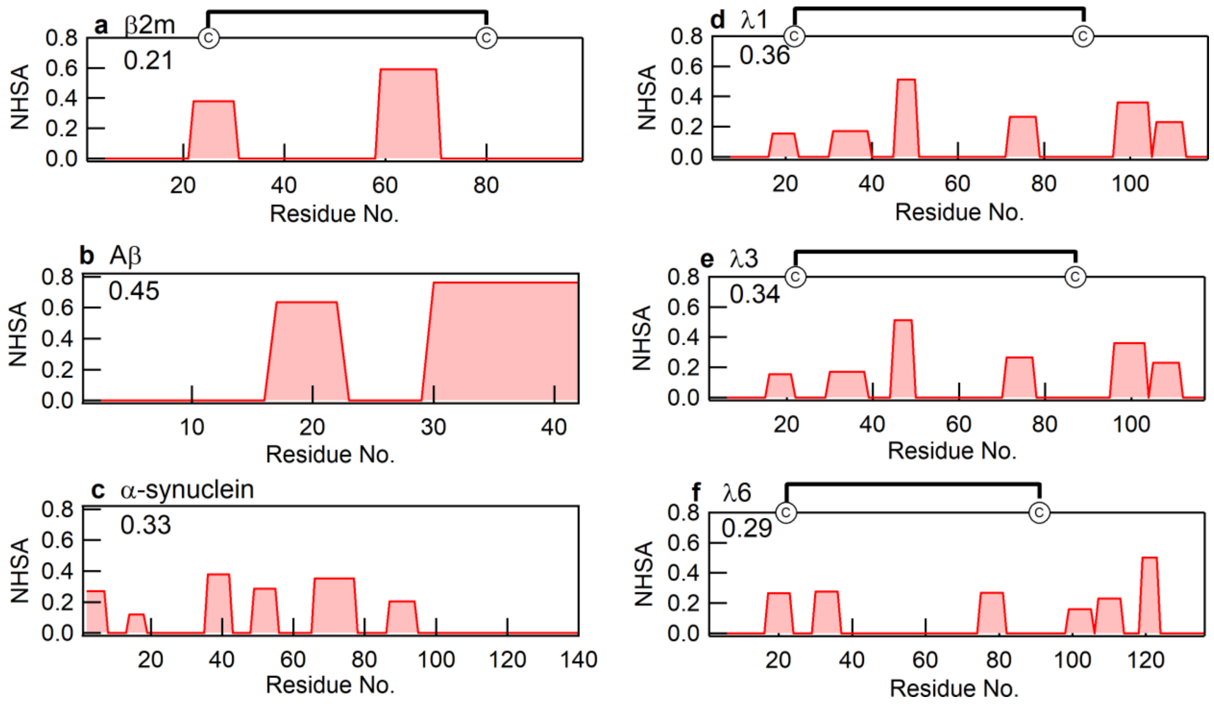


**Figure S6.** Predictions of aggregation-prone regions (APRs) by Aggrescan.[1]

The red lines indicate the normalized Hot-Spot Area provided by Aggrescan[1] for β2m (a), amyloid-β (b), α-synuclein (c), and the immunoglobulin λ1 (d), λ3 (e), and λ6 (f) light chains. The numbers inside the panel indicate the ratio of the number of APR residues with respect to the total number of residues. The positions of "C" on the top of the panels indicate the positions of the disulfide bond, if any.

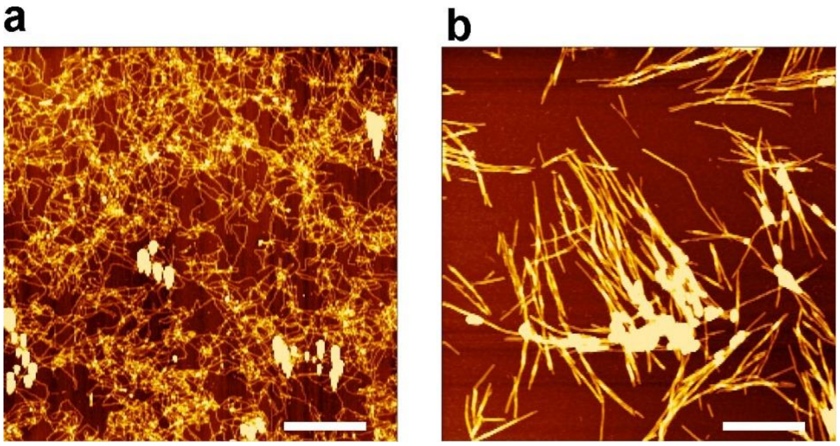


**Figure S7.** AFM images of amyloid fibrils of reduced (a) and oxidized (b) β2m.

The procedure of preparation of the reduced β2m sample was described in Material and Methods in the main text. The fibrils of both forms were prepared in 1.0 mg/mL β2m, 20 mM Gly (pH 2.5), 100 mM NaCl, 37°C for 3 days with contiguous stirring (10 second vortex/ 110 second quiescent). Scale bars represent 1 μm. These images were obtained using an AFM5100N (Hitachi High-Tech Science, Tokyo, Japan). The sample solution was diluted 10-fold with water, and 20 μL of solution was loaded to a mica plate for one minute, and then rinsed with 100 μL of water. Water was then absorbed by filter paper and the plate was dried. The sweep rate was set to 0.5 or 1.0 Hz.

**Supplementary Methods**

Deviation of $I_{para}/I_{dia}$ values for random coils

The ratio of the signal strength of the paramagnetic label sample to the signal strength of the diamagnetic reference was calculated by equation (1).

$$\frac{I_{para}}{I_{dia}} = \frac{R_{2,dia}\exp(-R_{2P}t)}{R_{2,dia}+R_{2P}} \qquad (1)$$

where $I_{para}$ and $I_{dia}$ represent the signal intensities of the paramagnetic-labeled sample and quenched sample (by an ascorbic acid treatment), respectively. $t$ is the length of the INEPT period of the HSQC measurement, which is 10 ms here. $R_{2,dia}$ are the transverse relaxation rate constants of each residue of the quenched sample, and are assumed to be 12.6 $s^{-1}$ (line width 4 Hz). $R_{2P}$ are the transverse relaxation rate constants of the paramagnetic-labeled sample, and are calculated by equation (2).[2]

$$R_{2P} = \frac{K}{r^6}\left(4\tau_c + \frac{3\tau_c}{1+\omega_{\mathrm{H}}^2\tau_{\mathrm{C}}^2}\right) \qquad (2)$$

where $r$ is the distance between the label and the NH bond of the amide group, and $K$ is the interaction constant between the unpaired electron and proton, which is $1.23 \times 10^{-32}$ $cm^6$ $s^{-2}$. $\omega_H$ is the Larmor frequency of hydrogen, which is 600 MHz. $\tau_C$ is the correlation time of electron-nuclear dipole-dipole interaction, assumed to be 4 ns. For $r$, Equation (3),[3] which is the relational expression between the radius of gyration ($R_g$) and the residue length ($N$) of the highly denatured protein, is used, and the distance between the ends of the polypeptide chain of the number of residues is substituted.

$$r = \sqrt{6}R_g = \sqrt{6}R_0N^{0.61} \qquad (3)$$

where $R_0$ is set to 1.82 Å.

**Table S1.** Polypeptide backbone $^{1}H$, $^{13}C$, and $^{15}N$ chemical shifts for β2m in the acid-denatured state at 20 mM Gly-HCl (pH 2.5) and 25°C. Chemical shifts indicated by ? were not obtained unambiguously. The chemical shifts of $^{1}H$ and $^{15}N$ for the amide groups shown in red indicate that these values were obtained not from the sequential assignments, but from estimations of chemical shift similarities with those from no salt conditions.

| | | Oxidized form | | | | | | | Reduced form | | | | | |
|---|---|---|---|---|---|---|---|---|---|---|---|---|---|---|
| #NUM | AA | HA | CA | CB | CO | N | HN | | HA | CA | CB | CO | N | HN |
| 0 | M | - | 58.34 | 36.19 | 172.1 | - | - | | - | 58.33 | 36.21 | 172.1 | - | - |
| 1 | I | 4.186 | 64.53 | 41.97 | 175.5 | 124.4 | 8.586 | | 4.206 | 64.49 | 41.98 | 175.5 | 124.4 | 8.596 |
| 2 | Q | 4.355 | 58.94 | 32.85 | 175.6 | 125.8 | 8.574 | | 4.369 | 58.91 | 32.91 | 175.6 | 125.8 | 8.587 |
| 3 | R | 4.396 | 59.43 | 34.21 | 176.1 | 123.9 | 8.527 | | 4.400 | 59.45 | 34.16 | 176.1 | 123.9 | 8.542 |
| 4 | T | 4.585 | 63.17 | 72.84 | 172.7 | 118.2 | 8.255 | | 4.597 | 63.14 | 72.83 | 172.7 | 118.2 | 8.263 |
| 5 | P | ? | 66.48 | 35.64 | 176.5 | ? | - | | ? | 66.33 | 35.76 | 175.6 | ? | - |
| 6 | K | 4.275 | 59.63 | 35.82 | ? | 122.1 | 8.363 | | 4.279 | 59.60 | 29.41 | ? | 122.1 | 8.372 |
| 7 | I | ? | 64.16 | 42.04 | 175.9 | 122.3 | 8.191 | | ? | 64.14 | 42.03 | 175.9 | ? | ? |
| 8 | Q | 4.331 | 58.79 | 32.89 | 175.3 | 125.5 | 8.448 | | 4.333 | 58.80 | 32.91 | 175.3 | 125.6 | 8.464 |
| 9 | V | 4.045 | 65.52 | 36.00 | 175.7 | 122.2 | 8.142 | | 4.058 | 65.50 | 36.15 | 175.7 | 122.3 | 8.155 |
| 10 | Y | 4.618 | 61.06 | 42.27 | 175.6 | 124.5 | 8.330 | | 4.625 | 61.01 | 42.27 | 175.6 | 124.6 | 8.356 |
| 11 | S | 4.386 | 61.45 | 67.27 | 174.0 | 118.0 | 8.179 | | 4.389 | 61.30 | 67.26 | 173.9 | 118.1 | 8.193 |
| 12 | R | 4.246 | 59.31 | 34.07 | 175.7 | 122.9 | 8.253 | | 4.265 | 59.36 | 34.05 | 175.7 | 122.9 | 8.270 |
| 13 | H | 4.963 | 56.27 | 31.64 | 172.2 | 120.1 | 8.466 | | 4.973 | 58.94 | 31.73 | 172.3 | 120.1 | 8.486 |
| 14 | P | ? | 66.60 | 35.43 | 176.5 | ? | - | | ? | 66.55 | 35.40 | 176.5 | ? | - |
| 15 | A | 4.294 | 55.99 | 22.41 | 177.8 | 124.6 | 8.483 | | 4.292 | 55.93 | 22.36 | 177.8 | 124.6 | 8.492 |
| 16 | E | 4.375 | 59.12 | 31.99 | 175.7 | 119.7 | 8.377 | | 4.381 | 58.98 | 32.42 | 175.8 | 119.9 | 8.395 |
| 17 | N | 4.703 | 56.34 | 42.12 | 175.7 | 119.9 | 8.458 | | 4.693 | 56.47 | 42.18 | 175.7 | 119.9 | 8.485 |
| 18 | G | 3.954 | 48.74 | - | 174.2 | 109.3 | 8.387 | | 3.959 | 48.80 | - | 174.2 | 109.3 | 8.403 |
| 19 | K | 4.339 | 59.76 | 36.24 | 176.7 | 120.6 | 8.150 | | 4.351 | 59.57 | 36.29 | 176.7 | 120.6 | 8.159 |
| 20 | S | 4.403 | 61.87 | 67.22 | 174.1 | 116.3 | 8.272 | | 4.407 | 61.88 | 67.14 | 174.2 | 116.5 | 8.302 |
| 21 | N | 4.668 | 56.44 | 42.18 | 174.8 | 120.6 | 8.329 | | 4.665 | 56.46 | 42.15 | 174.9 | 120.7 | 8.371 |
| 22 | F | 4.609 | 61.29 | 42.38 | 175.5 | 119.9 | 8.091 | | 4.599 | 61.35 | 42.60 | 175.6 | 120.2 | 8.122 |
| 23 | L | ? | ? | ? | ? | ? | ? | | 4.281 | 58.73 | 45.57 | 176.9 | 122.6 | 8.041 |
| 24 | N | ? | ? | ? | ? | 118.7 | 8.255 | | 4.618 | 56.64 | 41.95 | 175.0 | 119.0 | 8.259 |
| 25 | C | ? | ? | ? | ? | 118.9 | 8.168 | | 4.399 | 61.77 | 31.29 | 174.0 | 118.4 | 8.040 |
| 26 | Y | ? | ? | ? | ? | 121.3 | 8.302 | | 4.278 | 61.57 | 42.02 | 175.7 | 122.4 | 8.193 |
| 27 | V | ? | ? | ? | 175.8 | 121.0 | 7.947 | | 4.085 | 65.55 | 36.05 | 175.8 | 121.7 | 7.926 |

| | | | | | | | | | | | | | | |
|---|---|---|---|---|---|---|---|---|---|---|---|---|---|---|
| 28 | S | 4.603 | 61.89 | 67.26 | 174.8 | 118.7 | 8.254 | | 4.369 | 62.07 | 67.07 | 175.0 | 118.9 | 8.276 |
| 29 | G | 3.877 | 48.49 | - | 173.4 | 110.4 | 8.266 | | 3.906 | 48.41 | - | 173.4 | 110.6 | 8.300 |
| 30 | F | 4.476 | 60.79 | 42.93 | 174.8 | 120.1 | 7.968 | | 4.071 | 61.04 | 42.93 | 174.9 | 120.3 | 8.018 |
| 31 | H | ? | ? | ? | ? | 122.0 | 8.182 | | 4.833 | 55.97 | 31.73 | 171.6 | 122.5 | 8.230 |
| 32 | P | ? | 66.91 | 35.63 | 176.7 | ? | - | | ? | 66.73 | 35.31 | 176.8 | ? | - |
| 33 | S | 4.399 | 61.81 | 67.45 | 174.2 | 115.4 | 8.330 | | 4.418 | 61.93 | 67.24 | 174.4 | 115.6 | 8.393 |
| 34 | D | 4.702 | 56.32 | 41.71 | 175.2 | 120.3 | 8.407 | | 4.681 | 56.97 | 42.14 | 175.4 | 121.0 | 8.437 |
| 35 | I | 4.222 | 64.92 | ? | 175.8 | 119.6 | 7.916 | | 4.158 | 64.92 | 41.90 | 176.2 | 119.9 | 7.945 |
| 36 | E | ? | ? | ? | ? | 123.2 | 8.161 | | 4.328 | 59.67 | 32.13 | 176.3 | 123.6 | 8.240 |
| 37 | V | ? | ? | ? | ? | 120.4 | 8.112 | | 4.034 | 66.66 | 35.83 | 176.1 | 120.6 | 8.111 |
| 38 | D | ? | ? | ? | ? | 121.4 | 8.368 | | 4.695 | 56.90 | 42.26 | 175.7 | 121.7 | 8.402 |
| 39 | L | ? | ? | ? | ? | 121.8 | 8.087 | | 4.372 | 60.66 | 36.22 | 176.5 | 121.7 | 8.086 |
| 40 | L | ? | ? | ? | ? | 121.8 | 8.126 | | 4.081 | 58.87 | 36.16 | 178.0 | 121.4 | 8.088 |
| 41 | K | ? | ? | ? | ? | 120.5 | 8.201 | | 4.426 | 59.36 | 34.25 | 176.9 | 120.6 | 8.165 |
| 42 | N | ? | 57.20 | 41.90 | 175.9 | 118.4 | 8.289 | | 4.603 | 56.92 | 41.79 | 176.0 | 118.4 | 8.273 |
| 43 | G | 4.011 | 49.07 | - | 174.2 | 108.2 | 8.268 | | 3.944 | 49.05 | - | 174.2 | 108.4 | 8.287 |
| 44 | E | 4.331 | 59.39 | 32.08 | 175.7 | 119.6 | 8.013 | | 4.337 | 59.48 | 32.47 | 175.8 | 119.6 | 8.046 |
| 45 | R | ? | ? | ? | ? | 121.3 | 8.303 | | 4.417 | 59.48 | 34.27 | 176.2 | 121.7 | 8.290 |
| 46 | I | ? | ? | ? | ? | 121.9 | 8.269 | | ? | 64.76 | 42.20 | 133.1 | 122.0 | 8.225 |
| 47 | E | ? | ? | ? | ? | 123.5 | 8.415 | | 4.347 | 59.43 | 32.48 | 175.9 | 123.7 | 8.393 |
| 48 | K | ? | ? | ? | ? | 122.3 | 8.293 | | 4.333 | 59.72 | 36.23 | 176.3 | 122.4 | 8.295 |
| 49 | V | ? | 59.12 | 33.28 | 175.4 | 121.0 | 8.068 | | 4.071 | 65.57 | 36.12 | 176.0 | 121.0 | 8.125 |
| 50 | E | 4.411 | 59.24 | 32.43 | 175.7 | 124.3 | 8.622 | | 4.307 | 59.19 | 32.45 | 175.8 | 123.7 | 8.397 |
| 51 | H | 4.648 | 58.92 | 32.05 | 174.2 | 118.7 | 8.462 | | 4.700 | 58.98 | 32.12 | 174.2 | 119.0 | 8.485 |
| 52 | S | 4.440 | 61.82 | 67.40 | 174.1 | 115.9 | 8.247 | | 4.426 | 61.93 | 67.25 | 174.2 | 116.3 | 8.301 |
| 53 | D | 4.718 | 56.45 | 41.58 | 175.3 | 120.9 | 8.477 | | 4.692 | 56.42 | 42.09 | 175.7 | 121.3 | 8.491 |
| 54 | L | ? | 59.03 | 45.72 | 177.3 | 122.2 | 8.138 | | 4.657 | 58.98 | 45.38 | 177.5 | 122.6 | 8.133 |
| 55 | S | 4.336 | 61.88 | 66.87 | 174.4 | 115.5 | 8.089 | | 4.320 | 61.98 | 66.91 | 174.4 | 115.6 | 8.124 |
| 56 | F | 4.529 | 61.65 | 42.56 | 175.9 | 121.6 | 8.036 | | 4.555 | 61.71 | 42.55 | 175.9 | 121.5 | 8.019 |
| 57 | S | 4.239 | 59.60 | 67.00 | 175.7 | 115.6 | 7.981 | | 4.267 | 61.79 | 67.08 | 175.0 | 115.8 | 7.986 |
| 58 | K | 4.523 | 59.42 | 33.99 | 176.3 | 121.5 | 8.271 | | 4.221 | 60.70 | 35.79 | 176.6 | 122.2 | 8.194 |
| 59 | D | ? | ? | ? | ? | 118.1 | 8.211 | | 4.686 | 58.49 | 44.36 | 177.0 | 118.7 | 8.201 |
| 60 | W | ? | ? | ? | ? | 121.5 | 8.092 | | 4.105 | 62.05 | 32.75 | 176.7 | 121.4 | 8.092 |
| 61 | S | ? | ? | ? | ? | 115.1 | 8.105 | | 4.118 | 63.79 | 66.47 | 176.4 | 115.3 | 8.041 |
| 62 | F | ? | ? | ? | ? | 121.3 | 7.873 | | 4.363 | 62.99 | 42.37 | 176.2 | 121.3 | 7.786 |
| 63 | Y | ? | ? | ? | ? | 119.3 | 7.875 | | 4.248 | 63.55 | 43.09 | 176.2 | 119.1 | 7.822 |

| | | | | | | | | | | | | | | |
|---|---|---|---|---|---|---|---|---|---|---|---|---|---|---|
| 64 | L | ? | ? | ? | ? | 119.7 | 7.903 | | 4.189 | 59.83 | 45.62 | 178.1 | 119.4 | 7.876 |
| 65 | L | ? | ? | ? | ? | ? | ? | | 4.161 | ? | ? | 177.6 | 120.1 | 7.697 |
| 66 | Y | ? | ? | ? | ? | 118.8 | 7.850 | | 4.332 | ? | ? | 177.0 | 118.3 | 7.789 |
| 67 | Y | ? | ? | ? | ? | 118.9 | 8.064 | | 4.326 | 62.31 | 42.27 | 176.3 | 118.8 | 7.917 |
| 68 | T | ? | ? | ? | ? | ? | ? | | 4.221 | 65.75 | 72.91 | 174.6 | 112.8 | 7.821 |
| 69 | E | ? | ? | ? | ? | 120.0 | 7.966 | | 4.324 | 59.62 | 32.06 | 175.6 | 120.8 | 8.009 |
| 70 | F | ? | ? | ? | ? | 120.2 | 7.965 | | 4.703 | 60.47 | 42.95 | 175.6 | 120.5 | 7.986 |
| 71 | T | ? | ? | ? | ? | 116.8 | 8.018 | | 4.559 | 62.92 | 72.67 | 172.8 | 117.4 | 7.973 |
| 72 | P | ? | ? | ? | ? | ? | - | | ? | 67.47 | 35.34 | 177.4 | ? | - |
| 73 | T | ? | ? | ? | 175.3 | 111.6 | 8.025 | | 4.234 | 66.36 | 72.94 | 175.2 | 112.8 | 8.044 |
| 74 | E | ? | ? | ? | 176.7 | 121.4 | 7.985 | | 4.277 | 60.04 | 31.85 | 176.7 | 122.2 | 8.104 |
| 75 | K | ? | ? | ? | 176.9 | 120.1 | 8.283 | | 4.171 | 60.71 | 35.94 | 176.9 | 121.2 | 8.336 |
| 76 | D | ? | ? | ? | ? | 118.1 | 8.131 | | ? | 57.42 | 42.13 | 133.1 | 119.0 | 8.256 |
| 77 | E | ? | ? | ? | ? | 119.7 | 8.046 | | 4.324 | 60.12 | 31.78 | 176.4 | 120.4 | 8.159 |
| 78 | Y | ? | ? | ? | ? | 119.7 | 8.046 | | 4.464 | 61.85 | 41.76 | 175.9 | 119.6 | 8.082 |
| 79 | A | ? | ? | ? | ? | 122.6 | 7.937 | | 4.250 | 56.38 | 22.31 | 177.7 | 123.7 | 7.989 |
| 80 | C | ? | ? | ? | ? | 116.0 | 8.160 | | 4.428 | 62.29 | 31.05 | 174.8 | 117.1 | 8.092 |
| 81 | R | ? | ? | ? | ? | 122.3 | 8.292 | | 4.327 | 59.87 | 34.05 | 176.2 | 122.9 | 8.213 |
| 82 | V | ? | ? | ? | ? | 120.6 | 8.120 | | 4.521 | 65.90 | 35.83 | 176.2 | 120.2 | 8.016 |
| 83 | N | ? | 56.58 | 42.53 | 174.6 | 122.1 | 8.380 | | 4.679 | 56.61 | 42.27 | 174.7 | 122.1 | 8.373 |
| 84 | H | 4.689 | 58.62 | 32.19 | 173.9 | 119.1 | 8.420 | | 4.693 | 58.71 | 32.10 | 174.0 | 119.1 | 8.418 |
| 85 | V | 4.151 | 65.76 | 36.16 | 175.9 | 121.9 | 8.193 | | 4.141 | 65.60 | 36.15 | 176.0 | 121.8 | 8.206 |
| 86 | T | 4.361 | 65.36 | 73.09 | 174.2 | 119.5 | 8.319 | | 4.343 | 65.38 | 73.09 | 174.2 | 119.6 | 8.342 |
| 87 | L | 4.401 | 58.37 | 45.86 | 177.0 | 125.7 | 8.346 | | 4.398 | 58.44 | 45.75 | 177.1 | 125.7 | 8.357 |
| 88 | S | 4.443 | 61.53 | 67.21 | 173.9 | 116.9 | 8.257 | | 4.438 | 61.54 | 67.16 | 173.9 | 117.0 | 8.276 |
| 89 | Q | 4.607 | 56.83 | 32.14 | 173.8 | 123.0 | 8.333 | | 4.618 | 56.77 | 32.14 | ? | 123.1 | 8.335 |
| 90 | P | ? | 66.49 | 35.70 | 176.6 | ? | - | | ? | 66.46 | 35.73 | 176.5 | ? | - |
| 91 | K | 4.275 | 59.75 | 35.77 | 176.4 | 121.5 | 8.343 | | 4.302 | 59.74 | 35.96 | 176.4 | 121.6 | 8.364 |
| 92 | I | 4.120 | 64.16 | 41.88 | 175.9 | 122.8 | 8.128 | | 4.148 | 64.19 | 41.95 | 175.9 | 122.9 | 8.142 |
| 93 | V | 4.037 | 65.59 | 35.98 | 175.7 | 125.2 | 8.210 | | 4.045 | 65.37 | 36.23 | 175.7 | 125.4 | 8.235 |
| 94 | K | 4.272 | 59.71 | 36.31 | 175.8 | 125.1 | 8.239 | | 4.287 | 59.38 | 34.69 | 175.8 | 125.2 | 8.244 |
| 95 | W | 4.137 | 60.12 | 32.95 | 175.7 | 122.8 | 8.129 | | ? | 60.22 | 33.04 | 175.7 | 122.9 | 8.140 |
| 96 | D | 4.599 | 56.11 | 41.59 | 174.7 | 121.0 | 8.181 | | 4.594 | 56.26 | 42.04 | 174.8 | 121.3 | 8.185 |
| 97 | R | 4.134 | 59.67 | 33.73 | 175.7 | 120.7 | 7.988 | | 4.141 | 59.70 | 33.77 | 175.7 | 120.6 | 7.987 |
| 98 | D | 4.686 | 56.40 | 41.37 | 174.7 | 119.1 | 8.297 | | 4.679 | 56.65 | 41.72 | 174.6 | 119.4 | 8.323 |
| 99 | M | 4.435 | 58.27 | 35.94 | 178.3 | 121.7 | 8.075 | | 4.388 | 58.82 | 36.19 | 178.9 | 122.6 | 8.003 |

**Table S2.** Polypeptide backbone $^{1}H$, $^{13}C$, and $^{15}N$ chemical shifts for $\beta_2$-microglobulin in the acid-denatured state at 4 mM HCl (pH 2.5) and 37°C reported by Katou et al.[4] Chemical shifts indicated by ? were not obtained unambiguously.

| | | Oxidized form | | | | | | | Reduced form | | | | | |
|---|---|---|---|---|---|---|---|---|---|---|---|---|---|---|
| #NUM | AA | HA | CA | CB | CO | N | HN | | HA | CA | CB | CO | N | HN |
| -4 | E | ? | ? | ? | ? | - | - | | ? | ? | ? | ? | - | - |
| -3 | A | 4.37 | ? | 19.44 | ? | 127.6 | 8.58 | | 4.36 | ? | ? | ? | 127.5 | 8.58 |
| -2 | Y | 4.61 | ? | 38.95 | ? | 121.2 | 8.15 | | 4.58 | ? | ? | ? | 121.2 | 8.15 |
| -1 | V | 4.08 | ? | 19.57 | ? | 123.9 | 7.98 | | 4.07 | ? | ? | ? | 123.9 | 7.97 |
| 1 | I | 4.09 | ? | ? | ? | 126.0 | 8.09 | | 4.07 | ? | ? | ? | 126.0 | 8.09 |
| 2 | Q | 4.36 | ? | ? | ? | 125.8 | 8.36 | | 4.35 | ? | ? | ? | 125.8 | 8.36 |
| 3 | R | 4.40 | ? | 30.90 | ? | 124.0 | 8.39 | | 4.39 | 55.65 | 30.88 | ? | 124.0 | 8.38 |
| 4 | T | ? | ? | 69.77 | ? | 118.3 | 8.12 | | 4.57 | 60.23 | 69.83 | ? | 118.3 | 8.11 |
| 5 | P | ? | 63.23 | 32.33 | 176.5 | - | - | | 4.40 | 63.23 | 32.35 | 176.5 | - | - |
| 6 | K | 4.29 | 56.45 | 33.05 | 176.4 | 122.4 | 8.27 | | 4.27 | 56.43 | ? | 176.4 | 122.4 | 8.26 |
| 7 | I | 4.35 | 60.92 | 38.94 | 175.9 | 123.0 | 8.01 | | 4.13 | 60.94 | 38.98 | 175.9 | 122.9 | 8.01 |
| 8 | Q | ? | 55.63 | 29.77 | 175.3 | 125.9 | 8.37 | | 4.35 | 55.62 | 29.79 | 174.7 | 125.9 | 8.36 |
| 9 | V | 4.07 | 62.24 | 33.04 | 175.7 | 122.5 | 8.04 | | 4.06 | 62.29 | 33.03 | 175.7 | 122.5 | 8.04 |
| 10 | Y | 4.36 | 57.70 | 39.08 | 175.7 | 124.8 | 8.23 | | 4.62 | 57.73 | 39.08 | 175.6 | 124.9 | 8.23 |
| 11 | S | 4.40 | 57.92 | 64.02 | 174.0 | 118.4 | 8.11 | | 4.38 | 58.2 | 64.05 | 174.0 | 118.4 | 8.10 |
| 12 | R | 4.28 | 56.15 | 30.95 | 175.8 | 123.4 | 8.17 | | 4.27 | 56.2 | 30.99 | 175.7 | 123.4 | 8.17 |
| 13 | H | 4.97 | ? | 28.67 | 170.8 | 120.6 | 8.40 | | 4.95 | 53.37 | 28.64 | ? | 120.7 | 8.39 |
| 14 | P | ? | 63.31 | 32.25 | 176.6 | - | - | | 4.40 | 63.25 | 32.29 | 176.6 | - | - |
| 15 | A | 4.33 | 52.67 | 19.27 | 177.8 | 125.2 | 8.40 | | 4.31 | 52.64 | 19.31 | 177.8 | 125.2 | 8.39 |
| 16 | E | 4.39 | 55.75 | 29.11 | ? | 120.3 | 8.30 | | 4.37 | 55.74 | ? | 175.8 | 120.4 | 8.29 |
| 17 | N | 4.73 | 53.42 | 39.10 | ? | 120.5 | 8.40 | | 4.70 | 53.42 | 39.10 | 175.7 | 120.5 | 8.40 |
| 18 | G | 3.97<br>3.97 | 45.65 | - | 174.2 | 110.0 | 8.33 | | 3.96<br>3.96 | 45.64 | - | 174.2 | 109.9 | 8.32 |
| 19 | K | 4.36 | 56.41 | 33.18 | 176.7 | 121.2 | 8.09 | | 4.29 | 56.37 | 33.17 | 176.8 | 121.2 | 8.08 |
| 20 | S | 4.41 | 58.43 | 63.95 | 174.2 | 117.0 | 8.21 | | 4.40 | 58.41 | 63.87 | 174.2 | 117.1 | 8.22 |
| 21 | N | 4.68 | 53.43 | 39.03 | 174.8 | 121.3 | 8.28 | | 4.66 | 53.42 | 38.95 | 174.9 | 121.2 | 8.29 |
| 22 | F | 4.59 | 57.94 | 39.56 | 175.4 | 120.6 | 8.04 | | 4.59 | 58.06 | 39.57 | 175.6 | 120.8 | 8.05 |
| 23 | L | 4.17 | 55.40 | 42.73 | 176.6 | 123.2 | 8.00 | | 4.28 | 55.43 | 42.53 | 176.9 | 123.3 | 7.96 |
| 24 | N | 4.73 | 53.48 | 39.08 | 174.9 | 119.5 | 8.18 | | 4.62 | 53.49 | 38.84 | 175.1 | 119.6 | 8.19 |

| | | | | | | | | | | | | | | |
|---|---|---|---|---|---|---|---|---|---|---|---|---|---|---|
| 25 | C | ? | 55.95 | 41.98 | 173.8 | 119.0 | 8.13 | | 4.41 | 55.82 | 41.20 | 174.1 | 119.1 | 7.98 |
| 26 | Y | ? | 57.84 | 39.05 | 175.5 | 122.0 | 8.19 | | 4.58 | 58.13 | 38.66 | 175.8 | 123.0 | 8.12 |
| 27 | V | 4.10 | 62.29 | 33.04 | 175.8 | 121.7 | 7.91 | | 4.08 | 62.37 | 33.03 | 175.9 | 122.1 | 7.84 |
| 28 | S | 4.41 | 58.71 | 63.97 | 174.9 | 119.2 | 8.17 | | 4.37 | 58.66 | 63.87 | 175.0 | 119.4 | 8.19 |
| 29 | G | 3.90<br>3.90 | 45.34 | - | 173.4 | 110.9 | 8.20 | | 3.90<br>3.90 | 45.25 | - | 173.5 | 111.2 | 8.22 |
| 30 | F | ? | 57.64 | 39.86 | 174.8 | 120.8 | 7.92 | | 4.53 | 57.63 | 39.79 | 175.0 | 120.9 | 7.96 |
| 31 | H | 4.83 | 52.84 | 28.96 | 170.3 | 122.5 | 8.11 | | 4.83 | 52.77 | 28.89 | ? | 123.0 | 8.15 |
| 32 | P | ? | 63.29 | 32.11 | 176.8 | - | - | | 4.31 | 63.06 | 32.17 | 176.8 | - | - |
| 33 | S | 4.42 | 58.67 | 64.02 | 174.5 | 115.9 | 8.26 | | 4.42 | 58.55 | 63.94 | 174.5 | 116.2 | 8.32 |
| 34 | D | 4.72 | 54.00 | 38.87 | 175.3 | 121.0 | 8.34 | | 4.71 | 53.65 | ? | 175.2 | 121.5 | 8.36 |
| 35 | I | 4.21 | 61.69 | 38.94 | 176.0 | 120.2 | 7.86 | | 4.16 | 61.59 | 38.88 | 176.2 | 120.6 | 7.88 |
| 36 | E | 4.35 | 56.45 | 29.02 | 176.2 | 123.8 | 8.16 | | 4.34 | 56.44 | ? | 176.3 | 124.2 | 8.19 |
| 37 | V | 4.07 | 63.35 | 32.66 | 176.0 | 120.9 | 8.04 | | 4.04 | 63.21 | 32.69 | 176.1 | 121.0 | 8.00 |
| 38 | D | ? | 53.86 | 38.85 | 175.5 | 122.0 | 8.34 | | 4.67 | 53.8 | ? | 175.4 | 122.2 | 8.33 |
| 39 | L | ? | 55.85 | 42.62 | 177.5 | ? | ? | | ? | 56 | 42.31 | 177.6 | ? | ? |
| 40 | L | ? | 55.67 | 42.42 | 178.1 | 122.2 | 8.03 | | 4.34 | 55.45 | 42.31 | 178.0 | 122.0 | 7.99 |
| 41 | K | 4.24 | 57.33 | 32.91 | 176.8 | 121.3 | 8.13 | | 4.25 | 57.2 | 32.96 | 176.8 | 121.4 | 8.07 |
| 42 | N | ? | 53.82 | 38.68 | 176.0 | 118.9 | 8.20 | | 4.65 | 53.73 | 38.72 | 176.0 | 119.1 | 8.21 |
| 43 | G | 3.99<br>3.82 | 45.96 | - | 174.3 | 109.0 | 8.23 | | 3.92<br>3.92 | 45.85 | - | 174.3 | 109.2 | 8.23 |
| 44 | E | ? | 56.35 | 29.11 | 175.8 | 120.2 | 7.98 | | 4.35 | 56.20 | ? | 175.9 | 120.2 | 8.00 |
| 45 | R | ? | 56.41 | 31.12 | 176.3 | 122.0 | 8.21 | | 4.40 | 56.45 | 30.92 | 176.2 | 122.4 | 8.22 |
| 46 | I | ? | 61.30 | 39.03 | 176.0 | 122.2 | 8.16 | | 4.17 | 61.31 | 39.06 | 176.1 | 122.5 | 8.11 |
| 47 | E | 4.38 | 56.02 | 29.27 | 175.9 | 124.0 | 8.31 | | 4.40 | 55.93 | ? | 175.8 | 124.6 | 8.31 |
| 48 | K | ? | 56.59 | 33.47 | 176.3 | 122.6 | 8.23 | | 4.34 | 56.42 | 33.30 | 176.3 | 123.2 | 8.22 |
| 49 | V | 4.12 | 62.49 | 33.02 | 176.2 | 121.5 | 8.01 | | 4.08 | 62.38 | ? | 176.1 | 121.9 | 8.02 |
| 50 | E | 4.34 | 56.00 | 29.27 | 175.9 | 123.9 | 8.30 | | 4.33 | 55.9 | ? | 175.8 | 124.4 | 8.31 |
| 51 | H | 4.68 | 55.78 | 28.98 | 174.3 | 119.3 | 8.40 | | 4.68 | 55.59 | 29.17 | 174.3 | 119.8 | 8.42 |
| 52 | S | 4.45 | 58.48 | 64.04 | 174.2 | 116.5 | 8.19 | | 4.45 | 58.45 | 64.07 | 174.2 | 117.1 | 8.24 |
| 53 | D | 4.72 | 53.54 | 38.79 | 175.4 | 121.5 | 8.42 | | 4.70 | 53.40 | ? | 175.4 | 121.9 | 8.44 |
| 54 | L | 4.31 | 55.77 | 42.38 | 177.4 | 122.8 | 8.07 | | 4.28 | 55.60 | 42.30 | 177.4 | 123.0 | 8.08 |
| 55 | S | 4.34 | 58.79 | 63.66 | 174.5 | 116.1 | 8.03 | | 4.31 | 58.68 | 63.68 | 174.4 | 116.3 | 8.04 |
| 56 | F | 4.56 | 58.43 | 39.39 | 176.0 | 122.1 | 7.95 | | 4.56 | 58.23 | 39.47 | 175.9 | 122.2 | 7.95 |
| 57 | S | 4.27 | 58.87 | 63.80 | 175.1 | 116.3 | 7.91 | | 4.27 | 58.86 | 63.81 | 174.9 | 116.6 | 7.91 |
| 58 | K | ? | 57.29 | 32.64 | 176.7 | 122.7 | 8.10 | | 4.21 | 57.10 | 32.60 | 176.6 | 122.9 | 8.10 |

| | | | | | | | | | | | | | | |
|---|---|---|---|---|---|---|---|---|---|---|---|---|---|---|
| 59 | D | 4.72 | 53.80 | 38.88 | 175.7 | 118.8 | 8.15 | | 4.69 | 53.51 | ? | 175.5 | 119.2 | 8.14 |
| 60 | W | ? | 58.64 | 29.74 | 176.7 | 122.0 | 7.99 | | 4.33 | 58.52 | 29.70 | 176.7 | 122.3 | 7.99 |
| 61 | S | 4.16 | 60.26 | 63.27 | 175.1 | 115.8 | 7.98 | | 4.13 | 59.84 | 63.25 | 175.0 | 116.0 | 7.94 |
| 62 | F | 4.40 | 59.62 | 39.44 | 176.1 | 121.8 | 7.76 | | 4.35 | 59.27 | 39.34 | 176.1 | 121.9 | 7.72 |
| 63 | Y | 4.31 | 59.76 | 38.78 | 176.2 | 119.6 | 7.83 | | 4.27 | 59.72 | 38.45 | 176.2 | 119.8 | 7.74 |
| 64 | L | 4.17 | 56.53 | 42.38 | 178.0 | 120.2 | 7.84 | | 4.09 | 56.43 | 42.29 | 177.9 | 120.5 | 7.74 |
| 65 | L | 4.17 | 56.43 | 42.48 | 177.6 | 120.8 | 7.71 | | 4.12 | 56.25 | 42.36 | 177.6 | 120.7 | 7.64 |
| 66 | Y | 4.36 | 58.93 | 39.00 | 176.1 | 119.1 | 7.80 | | 4.36 | 58.70 | 38.89 | 176.0 | 119.0 | 7.72 |
| 67 | Y | 4.38 | 59.09 | 38.96 | 176.4 | 119.2 | 7.93 | | 4.39 | 58.91 | 38.77 | 176.3 | 119.7 | 7.85 |
| 68 | T | 4.27 | 62.82 | 69.88 | 174.6 | 113.1 | 7.80 | | 4.22 | 62.59 | 69.79 | 174.5 | 113.8 | 7.76 |
| 69 | E | 4.29 | 56.21 | 29.14 | 175.6 | 121.1 | 7.95 | | 4.28 | 55.99 | ? | 175.6 | 121.6 | 7.94 |
| 70 | F | ? | 57.34 | 39.79 | 175.5 | 121.2 | 7.92 | | 4.69 | 57.46 | 39.75 | 175.5 | 121.0 | 7.95 |
| 71 | T | 4.58 | 59.78 | 69.63 | 171.5 | 117.4 | 7.88 | | 4.55 | 59.77 | 69.69 | ? | 118.3 | 7.88 |
| 72 | P | ? | 64.24 | 32.11 | 177.4 | - | - | | 4.42 | 63.95 | 32.11 | 177.4 | - | - |
| 73 | T | 4.27 | 62.65 | 69.73 | 175.2 | 112.1 | 7.86 | | 4.25 | 62.64 | 69.71 | 175.1 | 113.4 | 7.96 |
| 74 | E | 4.26 | 56.88 | 28.82 | 176.6 | 122.1 | 7.97 | | 4.31 | 55.99 | ? | 176.5 | 122.8 | 8.07 |
| 75 | K | 4.16 | 57.85 | 32.76 | 177.0 | 120.9 | 8.19 | | 4.18 | 57.10 | 32.90 | 176.8 | 121.9 | 8.23 |
| 76 | D | ? | 54.01 | 38.56 | 175.5 | 118.7 | 8.12 | | 4.64 | 53.8 | ? | 175.6 | 119.6 | 8.21 |
| 77 | E | ? | 56.87 | 28.97 | 176.0 | 120.2 | 7.99 | | 4.24 | 56.61 | ? | 176.2 | 121.1 | 8.1 |
| 78 | Y | ? | 58.26 | 38.95 | 175.6 | 120.1 | 8.00 | | 4.46 | 58.35 | 38.57 | 175.9 | 120.3 | 7.99 |
| 79 | A | 4.35 | 52.84 | 19.59 | 177.5 | 124 | 7.98 | | 4.26 | 53.02 | 19.28 | 177.6 | 124.6 | 7.93 |
| 80 | C | 4.68 | 55.91 | 41.67 | 174.4 | 117.5 | 8.14 | | 4.43 | 56.06 | 40.99 | 174.7 | 117.9 | 8.03 |
| 81 | R | 4.41 | 56.31 | 31.11 | 175.9 | 122.7 | 8.23 | | 4.35 | 56.72 | 30.82 | 176.2 | 123.6 | 8.18 |
| 82 | V | 4.08 | 62.32 | 32.84 | 175.6 | 121.2 | 8.03 | | 4.06 | 62.35 | 32.93 | 175.6 | 120.9 | 7.94 |
| 83 | N | 4.70 | 53.23 | 39.31 | 174.7 | 122.9 | 8.33 | | 4.67 | 53.21 | 39.19 | 174.8 | 122.8 | 8.32 |
| 84 | H | 4.72 | 55.51 | 29.15 | 174.0 | 119.8 | 8.37 | | 4.70 | 55.45 | 29.11 | 174.1 | 119.9 | 8.36 |
| 85 | V | 4.17 | 62.52 | 33.06 | 176.0 | 122.4 | 8.13 | | 4.17 | 62.56 | 33.05 | 176.1 | 122.4 | 8.13 |
| 86 | T | ? | 62.01 | 69.91 | 174.2 | 120.0 | 8.24 | | 4.34 | 61.93 | 69.82 | 174.2 | 112.0 | 8.24 |
| 87 | L | 4.42 | 55.18 | 42.68 | 177.0 | 126.3 | 8.26 | | 4.40 | 55.19 | 42.69 | 177.1 | 126.3 | 8.25 |
| 88 | S | 4.45 | 58.22 | 63.94 | 174.0 | 117.6 | 8.19 | | 4.44 | 58.2 | 63.89 | 174.0 | 117.6 | 8.19 |
| 89 | Q | 4.64 | 53.91 | 29.27 | 172.4 | 123.7 | 8.26 | | 4.63 | 53.86 | 29.26 | ? | 123.7 | 8.24 |
| 90 | P | ? | 63.33 | 32.22 | 176.6 | - | - | | 4.39 | 63.24 | 32.49 | 176.6 | - | - |
| 91 | K | 4.29 | 56.45 | 33.07 | 176.3 | 122.0 | 8.26 | | 4.28 | 56.55 | ? | 176.3 | 122.1 | 8.27 |
| 92 | I | 4.17 | 60.93 | 38.89 | 176.0 | 123.2 | 8.03 | | 4.16 | 60.95 | 38.72 | 176.0 | 123.2 | 8.02 |
| 93 | V | 4.07 | 62.05 | 33.04 | 175.7 | 125.6 | 8.12 | | 4.05 | 62.1 | 33.07 | 175.7 | 125.6 | 8.12 |
| 94 | K | 4.27 | 56.34 | 33.14 | 175.8 | 125.5 | 8.15 | | 4.26 | 56.36 | 33.21 | 175.8 | 125.6 | 8.14 |

| | | | | | | | | | | | | | | |
|---|---|---|---|---|---|---|---|---|---|---|---|---|---|---|
| 95 | W | ? | 57.12 | 29.76 | 175.8 | 123.0 | 8.02 | | 4.67 | 57.12 | 29.8 | 175.8 | 123.0 | 8.01 |
| 96 | D | 4.64 | 53.01 | 38.74 | 174.8 | 121.6 | 8.14 | | 4.6 | 53.08 | ? | 174.8 | 121.7 | 8.13 |
| 97 | R | ? | 56.43 | 30.64 | 175.7 | 121.2 | 7.94 | | 4.17 | 56.51 | 30.75 | 175.7 | 121.2 | 7.92 |
| 98 | D | ? | 53.33 | 38.45 | 174.7 | 119.8 | 8.25 | | 4.68 | 53.28 | ? | 175.3 | 120.0 | 8.26 |
| 99 | M | ? | 55.48 | 33.15 | - | 122.8 | 8.01 | | 4.41 | ? | 33.18 | - | 122.8 | 7.98 |

**References**


1 Conchillo-Sole O, de Groot NS, Aviles FX, Vendrell J, Daura X, Ventura S. AGGRESCAN: a server for the prediction and evaluation of "hot spots" of aggregation in polypeptides. BMC Bioinformatics. 2007; 8: 65.

2 Teilum K, Kragelund BB, Poulsen FM. Transient structure formation in unfolded acyl-coenzyme A-binding protein observed by site-directed spin labelling. J Mol Biol. 2002; 324: 349-357.

3 Wilkins DK, Grimshaw SB, Receveur V, Dobson CM, Jones JA, Smith LJ. Hydrodynamic radii of native and denatured proteins measured by pulse field gradient NMR techniques. Biochemistry. 1999; 38: 16424-16431.

4 Katou H, Kanno T, Hoshino M, et al. The role of disulfide bond in the amyloidogenic state of $\beta_2$-microglobulin studied by heteronuclear NMR. Protein Sci. 2002; 11: 2218-2229.
